# NemaCapsules: microfluidics-integrated biocells for investigating *C. elegans* host-microbiome interactions in spaceflight

**DOI:** 10.1038/s41526-026-00634-9

**Published:** 2026-07-23

**Authors:** Bushra Rahman, Atiyya P. Saroyia, Dana Blackburn, Audrey J. Parish, Nathaniel Szewczyk, Monica Driscoll, Buck S. Samuel, Siva A. Vanapalli

**Affiliations:** 1 Department of Chemical Engineering, Texas Tech University, Lubbock, TX USA; 2 Department of Molecular Virology and Microbiology and Alkek Center for Metagenomics and Microbiome Research, Baylor College of Medicine, Houston, TX USA; 3 Heritage College of Osteopathic Medicine, Ohio University, Athens, OH USA; 4 Department of Molecular Biology and Biochemistry, Rutgers, The State University of New Jersey, New Brunswick, NJ USA

**Keywords:** Biological techniques, Biotechnology, Microbiology

## Abstract

Spaceflight studies in humans show that microgravity alters the gut microbiome, posing a risk to health. Understanding how microbiomes and host genetics influence physiology is critical for long-duration missions. The low-cost spaceflight model *C. elegans* provides a suitable means to study host-microbiome interactions. However, whole-organism measurements are limited by lacking suitable flight-ready hardware that enables long-term nematode culture compatible with on-orbit imaging. These limitations were addressed through development of NemaCapsules— microfluidics-integrated biocells, for spaceflight assessment of *C. elegans* locomotion under defined microbiome conditions, with minimal astronaut workload. This provided a fully sealed, gas-permeable system with media reservoir, enabling passive, long-term nematode culturing. NemaCapsules were evaluated across four host strains and three model microbiomes. Multi-day worm viability studies demonstrated high resilience across diverse host strains and microbiomes except for *daf-16* mutants. Crawling and swimming studies of worms with model microbiomes of *C. elegans* natural microbiota exhibited distinct locomotory activity from those exposed to *E. coli* OP50. Wild-type N2 generally exhibited the highest activity, followed by wild isolate strains; *daf-16* mutants demonstrated lowest activity. Compared to crawling, swimming better revealed differences arising from host-microbiome interactions. NemaCapsules provide a modular solution for on-orbit, multi-functional readouts in *C. elegans* with minimal crew intervention.

## Introduction

The gut microbiome plays a crucial role in host physiology, development, and disease susceptibility^1^, since the naturally occurring microbiota play roles in immune system regulation, epithelial protection, vascular development, and supply/synthesis of vitamins^2^. Disruptions in the gut microbial balance have been linked to a wide range of health issues at the gut site of origin, but also remotely in the brain, liver, and pancreas^2^. In spaceflight, alterations to the crew gut microbial composition were observed in several microbial genera before, during, and after a mission to the International Space Station (ISS)^3^. During spaceflight, such changes in microbiome composition may impact whole individual physiology. Several potential targets of the microbiome, such as immunity, bone health, and insulin signaling^2^, have already been identified as a concern for astronaut health. Thus, it is crucial to develop an understanding of the long-term impact of the gut microbiome in spaceflight, especially for long-duration missions.

Notably, host responses to the gut microbiome reported in spaceflight can vary. For example, one such study reported significant differences among 17 astronauts in viral-loading dynamics and microbiome composition over time^4^. Another study showed that the gastrointestinal tract microbiome of one crewmember was already more diverse than the others before flight and did not significantly change during a long-duration mission^3^. These patterns are consistent with the concept that the host genetic background influences the spaceflight impact on the microbiome. However, the small sample sizes and the confounding factors in human studies (e.g., stress, disrupted sleep, diet) limit understanding of how microgravity directly impacts physiological health and potential microbiome restructuring. Thus, microbiome investigations in spaceflight need models in which the host genotype can be systematically varied alongside defined gut-microbial communities.

While human and other mammalian models have provided insights, the costly nature of these systems plus genetically heterogeneous populations and microbiomes render them unsuitable for high-throughput studies. *Caenorhabditis elegans* has emerged as a model system due to its high-throughput, rapid generation times, large sample sizes^5–12^, and measurable whole-organismal interactions among different tissue systems. Importantly, *C. elegans* are also well suited for gut microbiome investigations, given their extensively mapped microbiota^13^, and techniques for axenic culture that allow controlled gut microbiota composition using single-species bacteria or multi-membered communities^12,14^.

The *C. elegans* gastrointestinal tract shares extensive morphological and functional similarities with humans. The intestine has structurally similar microvilli harboring highly conserved proteins that mediate shared functions of nutrient absorption, peristalsis, and protection against pathogens^15^. The well-characterized microbiota for *C. elegans* further enhances the utility of the model for spaceflight investigations^16^. Also advantageous is that *C. elegans* natural variant isolates from the wild have been collected from habitats all over the globe, yielding a wide repository of genetically diverse strains^17^, routinely used in genetics- and microbiome-based studies^18,19^. A model microbiome, BIGbiome (a 63-member community), was recently established to represent phylogenetic and functional diversity of the natural *C. elegans* microbiome^19^. In sum, available resources, coupled with an exceptionally well-defined and easily manipulated host system, are poised to accelerate host-microbiome insights in response to microgravity in ways that can inspire translational benefits.

Past successful spaceflight studies have utilized *C. elegans* for insights on stress responses, neuronal morphology, and muscular atrophy^11^, and have largely focused on mapping molecular changes in response to microgravity^20–27^. Whole-organism functional measurements have been relatively rare due to lack of flight-ready hardware for nematode culture that is compatible with imaging equipment available on ISS. We have recently shown that microfluidics-integrated hardware can be useful to address this gap and demonstrated *C. elegans* muscle strength declines in spaceflight^28,29^. However, multi-day imaging with minimal crew time remains a challenge due to lack of long-term nematode culture. Moreover, whereas the *C. elegans* gut microbiome has been comprehensively studied on Earth^30–32^, to date there have not been any studies to connect spaceflight-induced shifts in microbiome composition to whole-organism outcomes. The key to addressing these gaps in understanding is to conduct on-orbit imaging under conditions in which the microbiome can be manipulated. Microfluidics-based worm cultures^33,34^ can address both limitations. Microfluidic devices enable the microenvironment to be manipulated to change culture conditions in the context of chip life that offers an optically clear, fixed plane for high-quality imaging in real-time.

To be able to generate insights on the spaceflight impact of microbiome and genetic diversity on the whole organism, we developed a microfluidics-integrated hardware with the following considerations in mind: (1) *C. elegans* maintenance that requires minimum crew effort; (2) robust culture viability over time to enable microbial colonization of *C. elegans* gut; (3) liquid filled and sealed containment to ensure safe handling; and (4) high quality multi-day imaging of locomotory activity. To fulfill these criteria, we developed an integrated hardware termed “NemaCapsules” where microfluidics-based passive nematode culture (PNC) chambers^35^ are encased in a bio-cell (designed by BioServe Space Technologies) that contains media and is gas-permeable. NemaCapsules are a spaceflight-compliant, modular hardware with minimal crew requirements and meant to sustain long-term cultures of *C. elegans* for investigating host-microbiome interactions in spaceflight. The flexibility of altering the PNC chamber design within the NemaCapsules also enables multiplexed assessment of both crawling and swimming activity for each host/gut microbiome condition.

The objective of these studies, following the development and ground-validation of the platform, was also to test whether it can distinguish between functional viability and locomotion phenotypes across host/microbiome conditions. To validate the use of NemaCapsules for host-microbiome studies in spaceflight, we first conducted microfluidics testing, followed by hardware integration and optimization. We conducted worm viability and locomotory studies to assess host and microbiome impact on physiology. The host strains consisted of 4 sterile *C. elegans* strains: WT(N2), *daf-16/FOXO* mutant, ED3017, and JU1400. The latter two are wild isolates that test robustness of responses in genetically diverse backgrounds^19,36,37^. All strains were also genetically engineered to include auxin-inducible systems for developmental degradation of an essential sperm protein for prevention of progeny production^38,39^. We tested three microbiomes: *Escherichia coli* OP50, BIGbiome^19^, and a simplified 3-member community (3-biome; consisting of dominant colonizers of the BIGbiome community). The following results discussed are from ground testing on Earth to ensure these host and microbiome conditions can be effectively assessed in future ISS experiments.

## Results

### Development of NemaCapsules hardware for *C. elegans* spaceflight studies

The ultimate goal for development of NemaCapsules is to facilitate robust spaceflight experiments on the International Space Station (ISS) that allow for on-orbit real-time quantification of microbiome impacts on host locomotory activity. In contrast to ground experiments, there are special considerations and constraints for spaceflight studies. Successful experiments must therefore consider limits on crew time and intervention, long-term maintenance of filled and sealed culture vessels, on-board crew safety, and ease of operational protocols for the crew to conduct experiments. Specifically, a NemaCapsule unit consists of: (1) microfluidic crawling and swimming chambers for *C. elegans* - passive nematode culturing chambers (PNCs)^35^, and (2) a spaceflight-certified biocell, designed by BioServe Space Technologies, to provide structural support, a reservoir to contain liquid media with gas permeability, and a closed system. Thus, each NemaCapsule unit is a defined host-microbiome experimental condition, and multiple units can be availed based on the study design and requirements.

**Fig. 1:**
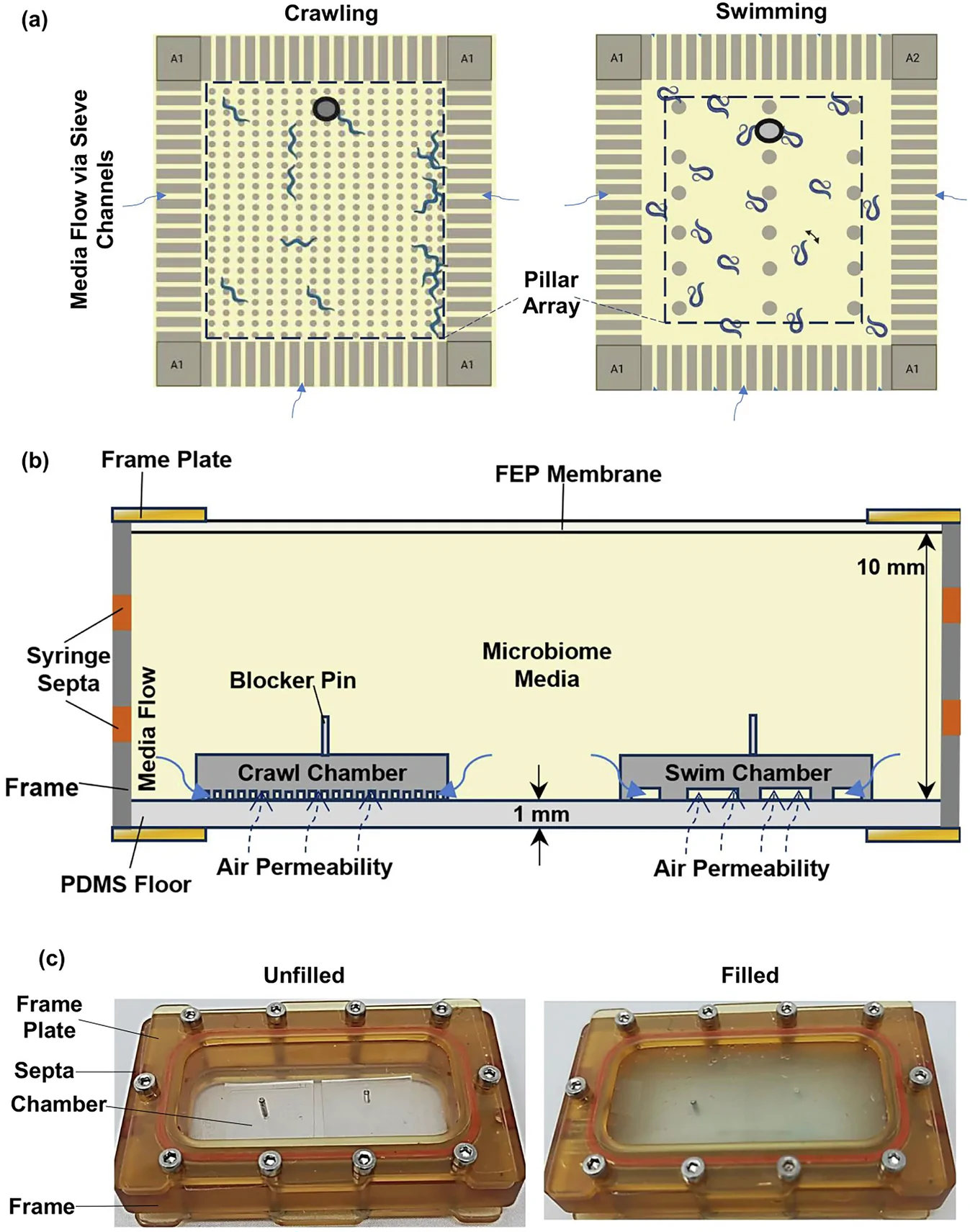
NemaCapsules unit and design elements. **a** Crawling PNC chamber (left) and swimming PNC chamber (right) each housed in a single NemaCapsules unit, with arrows representing passive media flow into the arena. Adapted from Rahman et al. (2026) *Lab on a Chip*. **b** Cross-sectional view of a single NemaCapsules unit consisting of a crawling chamber (left) and swimming chamber (right), bonded to the air permeable PDMS floor. The bulk space is filled with the microbiome media, through a syringe inserted into the septa. The media in the bulk flows towards the sieve openings of the chambers. The sieve and micropillar architecture for each of the chambers are shown in the insets, respectively, with crawling and swimming animals. **c** Images of fully assembled, unfilled (left) and microbiome media-filled (right) NemaCapsules units.

Microfluidic PNC chambers are the core components to house and observe the worms. The microenvironments were designed as 15 × 15 mm^2^ micropillar-arenas for culture that could enable high-quality image acquisition through a confined, optically clear plane. The crucial architecture of this design is the sieve channels (width: 35 µm) formed from the narrow gaps between the barriers (length: 1000 µm, width: 200 µm) for keeping adult worms inside (Fig. 1a). These channels facilitate continuous flow of media from the bulk space inside the chamber interior, where the worms are located. Thus, throughout the duration of the experiment, worms will have continuous access to their diet, without any feeding intervention required. In the context of spaceflight studies, this construction alleviates the potential feeding burden imposed on the crew for multi-day investigational payloads.

Additionally, the arena was designed with pillar spacing to facilitate worm movement throughout the footprint. With a diameter of 70 µm and edge-to-edge spacing of 90 µm, the pillar microarray was customized to help worms modulate a crawling gait. To complement crawling worms, we also designed a swimming PNC chamber with a similar architecture, but distinct pillar spacing. The PNC chamber was specifically adapted for swimming by fine-tuning the pillar array to a sparser distribution (350 µm diameter pillars, 750 µm edge-to-edge spacing) to allow native worm thrashing movements (Fig. 1a). Dual locomotory studies under the same physical environment and footprint offer an extended assessment of microbiome-induced variations, since the biology behind swimming arises from different neuromuscular activities compared to crawling^40,41^. Additionally, monitoring swimming provides a distinct visual health metric compared to crawling, given the literature-reported higher energy expenditure of worm movements when swimming^42^.

These chambers are housed within the same biocell, enabling paired recording and analysis under the same environmental conditions (Fig. 1b). Both chambers are bonded to a 1 mm-thick polydimethylsiloxane (PDMS) sheet custom-fitted to the biocell structure. The bonded microfluidic chambers are integrated into biocells (Fig. 1b,c), with the PDMS sheet screwed into place on both the frame and frame plate. To create a closed system, a fluorinated ethylene propylene (FEP) membrane is secured opposite the PDMS-bonded chamber. The combination of PDMS and FEP films is essential for maintaining worm cultures given their permeability to O_2_ and N_2_ gases. PDMS is an ideal choice for a sturdy microfluidic substrate and NemaCapsules floor, since the high diffusivity of PDMS adjacent to the worms provides them sufficient O_2_ exposure to thrive during the experimental period^43,44^. The >100-fold lower diffusivity of the FEP membrane^44^ is compensated through its 10X reduced sheet thickness compared to the PDMS floor, as well as its placement further away from the worm chamber locations. The structural support of the biocells helped to create a bulk space in the vessel, which was filled by microbiome media, for the worms’ diet (Fig. 1c). The microbiome was administered through insertion of the media-filled syringe through the septa. The assembly of dual microfluidic chambers and biocells culminates into modular NemaCapsules units, such that each capsule can be used to multiplex the crawling and swimming locomotory modes exhibited by a given worm host genotype and a defined microbiome.

### Validation and optimization of integrated crawling and swimming chambers

**Fig. 2:**
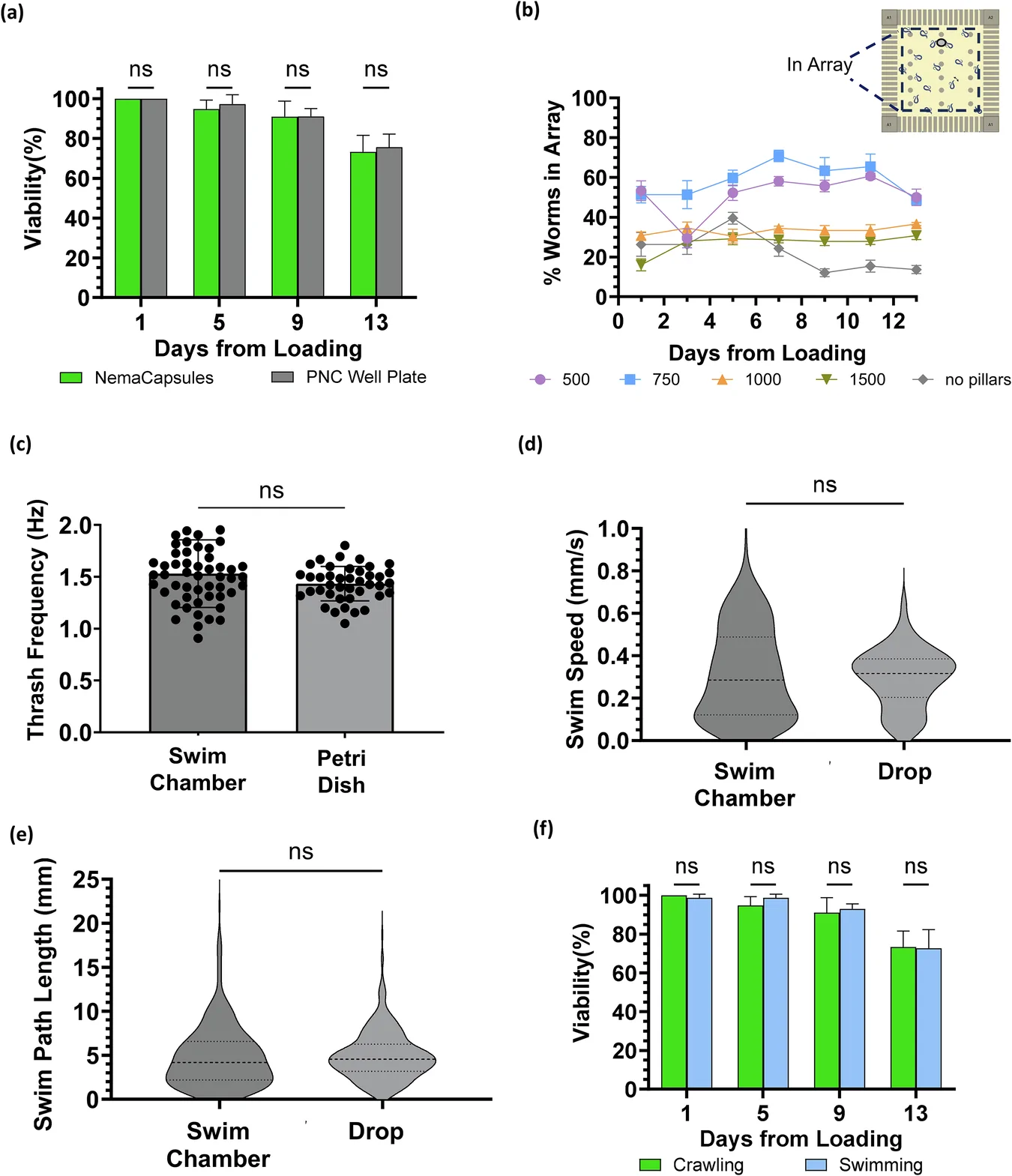
Testing and optimization of crawl and swim chambers. **a** Longitudinal viability data showing the direct comparability between PNCs integrated in NemaCapsules vs. multi-well plate PNCs to highlight adequate oxygen for worms in both platforms, with 2–3 PNC replicates per condition, 30–40 animals per chamber. Statistics conducted based on PNC replicates (*p* = 0.911 to 0.958, two-tailed *t*-tests). **b** Optimization of swim-pillar spacing in µm, with 2–3 microfluidic device replicates per condition, 30–40 animals per chamber. **c** Benchmarking in NemaCapsule (NC) swim chamber (750 µm spacing) to a Petri dish control (3 Petri dishes, 30–40 worms each) with day 2 adult WT AID worms in M9 media for both conditions. Statistics conducted based on PNC replicates (*p* = 0.0894, two-tailed *t*-tests). **d** The benchmarking from **b** also shown through speed of worms in swim chambers vs. petri dishes. Statistics conducted based on PNC replicates (*p* = 0.0943, two-tailed *t*-tests). **e** The benchmarking from **b** also shown through path length of worms in swim chambers vs. Petri dishes. Statistics conducted based on PNC replicates (*p* = 0.564, two-tailed *t*-tests). **f** Longitudinal viability data showing direct comparability between swim & crawl chambers, with 2–3 microfluidic device replicates per condition, 30–40 animals per chamber. Statistics conducted based on PNC replicates (*p* = 0.921 to 0.999, two-tailed *t*-tests).

To assess whether the performance of the closed system NemaCapsules is comparable to the established PNC well plates^35^ with an aerated system, we compared worm viability in both, as shown in Fig. 2a. The worms were WT(N2) and fed *E. coli* OP50 media. We examined viability from days 1 to 13 after loading in crawling chambers, with adults loaded at day 0 after being transferred from their auxin progeny-blocking treatment. A NemaCapsule unit was filled with 7 mL of media, whereas each PNC chamber in the well plates was surrounded by 2-mL of media per well to leave ample air headspace. Accordingly, *C. elegans* worms in NemaCapsules maintained viability comparable to multi-well plates over time, indicating that the closed architecture with the FEP/PDMS membranes in the NemaCapsules facilitates adequate oxygenation during cultivation (Fig. 2a).

To allow for assessment of swimming ability in parallel to crawling speed measurement, the micropillar arena for a second PNC chamber was reconfigured to a sparse pillar array (diameter ≈ 350 µm, edge-to-edge distance ≈ 750 µm). In contrast to a pillar-free design, sparse pillars safeguard against the potential caving in the empty chamber space^45,46^, allowing nematodes to move in an efficient manner^47,48^. The sparse pillar configuration was developed to minimize physical interactions and clustering along the arena edges, to ensure that worms remain within the arena center to ensure natural swimming patterns and high imaging quality for downstream video analyzes.

To optimize these parameters, we systematically evaluated the inter-pillar spacings (with a fixed pillar diameter of 350 µm) based on the percentage of worms (auxin-treated WT(N2)) navigating through the array over 13 days (Fig. 2b). The worms were imaged while *E. coli* OP50 bacteria remained in the devices and therefore had continuous access to food. We optimized pillar spacing through examining the % of worms traversing through the pillared array across varying inter-pillar spacings, as shown in the inset schematic of Fig. 2b. The worms counted were those moving within the outlined box surrounding the pillar array, rather than those only remaining in the peripheral barrier and sieve channel areas. The data shown in Fig. 2b reveal distinct trends. The tightest spacing, 500 µm, showed the second highest occupancy except for a downward spike on day 3 from loading. The 750 µm spacing demonstrated the highest occupancy, showing a consistent and sustained increase in the % worms utilizing the array, peaking at ~80% on day 7 from loading. At 1000 and 1500 µm spacings, the % worms navigating the array was lower compared to the 750 µm spacing (Fig. 2b). The sparser arrangements likely reduced structural guidance, leading to an increased tendency for worms to drift toward and linger at the barrier walls. In the absence of pillars, the proportion of worms exploring the non-peripheral area was the lowest through most of the study.

Without structural features, worms primarily accumulated along the edges and corners, resulting in clustering within the chamber—thus more difficulty in clearly counting worms for viability scoring. Based on these observations, we selected the 750 µm spacing as the optimal configuration for the subsequent experiments, striking the best balance between providing sufficient structure to guide locomotion while preserving natural swimming dynamics.

Concurrently, it was also important to establish that the chosen microfluidic pillar environment demonstrates that the swimming worms exhibit similar locomotory behavior compared to those in open-fluid environments. We conducted the benchmarking by comparing the locomotory behavior observed in the closed NemaCapsule swim chamber (750 µm pillar spacing) against those in a petri dish that is open to air, enabling adequate gas transport (Fig. 2c–e). In the benchmarking, we counted thrashes (2 min videos in each video, 10 fps) in the petri dish (500 µL volume) and swim chambers with the worms swimming in clear M9 media without food approximately 1 h after loading. For each video, the thrashing was scored over three separate 15 s intervals. These no-food conditions were used for only the PNC chamber vs. petri dish validation studies to align the assay conditions, and the manual body-bend counting approach with previously published swimming benchmarks that also scored thrashes over short fixed time intervals^47,49^. The thrashing rate in both conditions and the prior studies, converted to Hz, ranged from 1.3 to 1.5 Hz across all conditions and fell in range with prior studies^47,49,50^ (Fig. 2c–e). Thrashing in the NemaCapsules swim chamber vs. the petri dish control did not show statistical significance (Fig. 2c; two-tailed *t*-test, *p* = 0.0894). The estimated mean difference was 0.096 Hz (−0.207 to 0.015 at 95% CI), with a small effect size (*η*^2^ = 0.031), despite the slightly lower numerical values for the Petri dish. Additionally, speed and path length of the swimming worms from the same videos were quantified using wrMTrck (Fig. 2d,e). Neither speed (Fig. 2d; *p* = 0.0943; mean difference = −0.0227 mm/s, 95% CI −0.0493 to 0.0039; *η*² = 0.004) nor path length (Fig. 2e; *p* = 0.56; mean difference = 0.28 mm, 95% CI −0.33 to 0.60; *η*² = 0.0005) showed a statistically significant difference between the swim chamber and petri dish conditions. Taken together, these results support that the swim chamber environment allows for worms to exhibit similar locomotory behavior to those swimming in open fluid environments.

Lastly, we assessed worm viability in both the crawling and swimming chambers in NemaCapsules. As before, WT(N2) worms were fed OP50 and monitored at select time points until 13 days after loading. We observed that the viability remained high, from 98 to 73% (2-way ANOVA, *p* > 0.90) between the crawling and swimming chambers. Together, our data validate that the NemaCapsule architecture does not significantly impact worm health (Fig. 2d) and equally supports both crawling and swim chambers for whole-organism functional assessment.

### NemaCapsules orientation studies in secondary containment

**Fig. 3:**
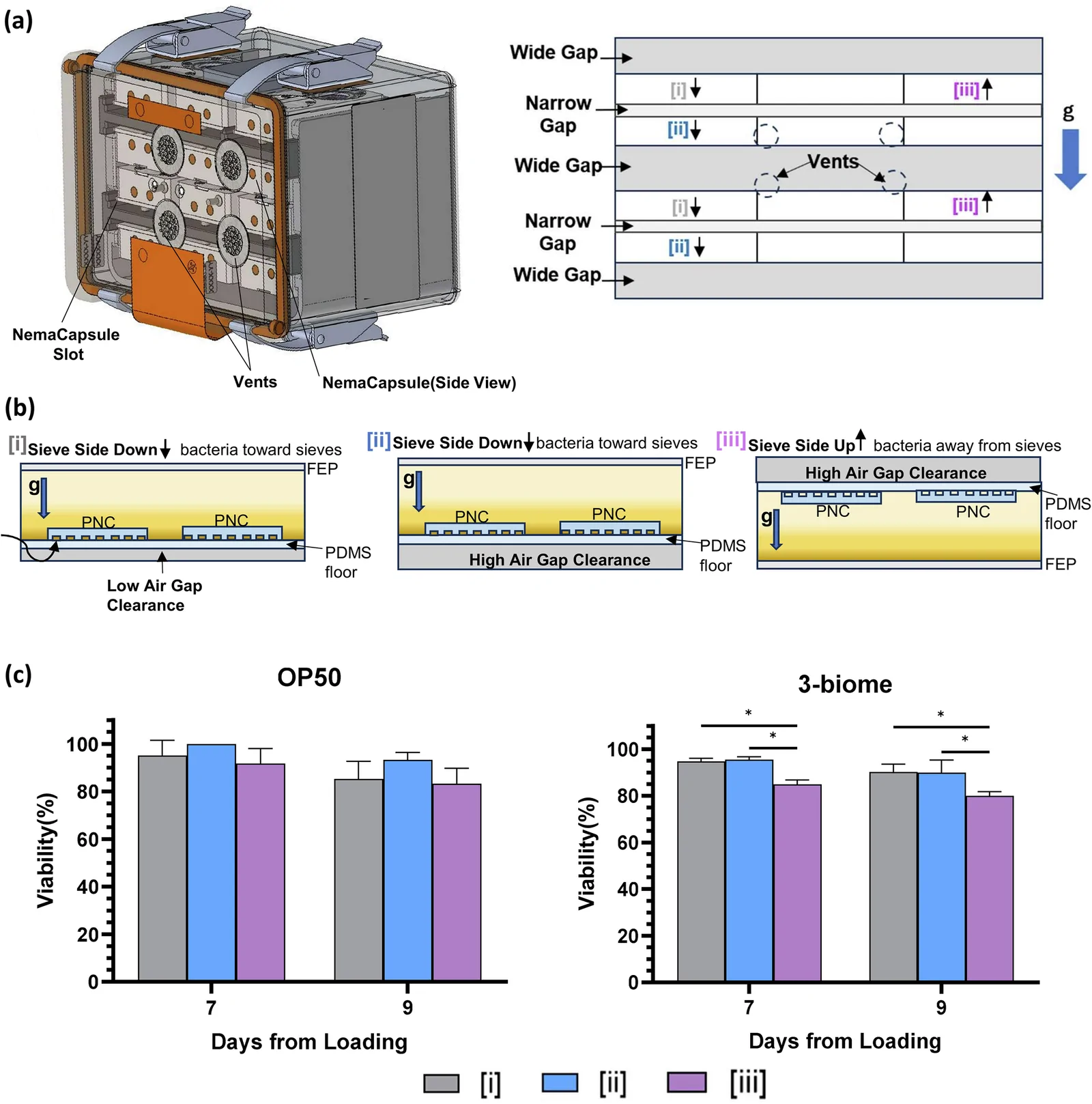
PHAB secondary containment for biocells. **a** 3-D rendering of NemaCapsules slotted securely in PHAB shelves (left) and cross-sectional schematic (right) inside PHAB highlighting areas of air clearance and labels designated to biocell configurations. **b** The three in-shelf biocell configurations ([i], [ii], and [iii]) displayed schematically. **c** Viability heatmaps comparing different PHAB shelf configurations amongst crawling WT(N2) animals. Sample sizes are as follows for OP50-fed worms: configuration [i] (*N* = 2 trials, *n* = 2 technical replicates, 88 animals); [ii] (*N* = 1 trial, *n* = 2 technical replicates, 87 animals); [iii] (*N* = 1 trials, *n* = 2 technical replicates, 82 animals). For 3-biome-fed worms: [i] (*N* = 2 trials, *n* = 2 technical replicates, 87 animals); [ii] (*N* = 1 trial, *n* = 2 technical replicates, 93 animals); [iii] (*N* = 1 trials, *n* = 2 technical replicates, 80 animals).

Spaceflight regulations require *C. elegans* and microbial suspensions within the NemaCapsules to be integrated into secondary containment to protect the ISS environment and limit crew member exposures. The Plate Habitat (PHAB) container is a flight-tested secondary containment system, developed by BioServe Space Technologies, that was selected to store the NemaCapsules during the non-imaging periods. Schematics of the PHAB layout and the viability outcomes from various biocell orientations are shown in Fig. 3. Briefly, the PHAB consists of racks, with rows of slots, custom-designed to hold the NemaCapsules units in place. Meshed vents are located on the front cover to ensure sufficient aeration inside the PHAB so the worms in the units receive adequate oxygenation. Top-to-bottom paired slots each consist of air gaps asymmetric to each other: one side with a narrower clearance to secure the unit, whereas the opposite side has a larger gap (Fig. 3a).

There are several challenges to overcome to integrate this system and apply it to spaceflight conditions. As the payloads remain in 1 g conditions for 1–2 days prior to microgravity exposure, the gravitational settling of bacterial suspensions becomes a key factor when determining the NemaCapsules orientation. From the final payload integration to launch, the NemaCapsules in the PHAB would remain in a fixed orientation under Earth gravity and cannot be agitated or re-homogenized. In this period, the bacterial suspension would settle downwards in the direction of gravity, effectively determining whether food accumulates towards or away from the sieve inlets of the PNC chambers, since the sieve inlets are the openings that facilitate media towards the worms, as described in section A.

To address these challenges, we considered several possible orientations of the PNC chambers when housed in the PHAB. Positioning the chambers downward, relative to the default orientation of the PHAB, promotes gravitational sedimentation of the bacterial suspension toward the sieve channels that the worms access. This sedimentation-favored orientation would help retain more bacteria in proximity to the worms. However, on the top shelf of the PHAB racks, this same position also places the PDMS floor against a narrow gap, potentially risking inadequate air exposure. Orienting the PNC chambers upward leads to bacteria settling away from the sieve region and the worms, but with the benefit of a wider gap (Fig. 3b).

When the PNC chambers are located in the upward position, the total time required for settling throughout the full height of the biocell H can be estimated as ${t}_{{\rm{set}}}=H/{V}_{s}$ where the Stokes terminal velocity, ${V}_{s}$ is the settling velocity due to gravitational sedimentation. Following Sun et al.^51^: ${V}_{s}=\frac{2{a}^{2}\varDelta \rho g}{9\mu }$. *a* is the effective radius of *E. coli OP50*, $\Delta \rho$ is the density difference between *E. coli OP50* and the liquid media, μ is dynamic viscosity. Using *a* = 0.5 μm^52^, Δ*ρ* = 100 kgm^−3^ (based on *E. coli* density of 1100 kgm^-3^^53^), $\mu =1.2\mathrm{mPa}.\mathrm{s}$, (consistent with standard water values and modest upward shifts reported for dilute aqueous salt and nutrient solutions^54^), and *H* = 9–10 mm, we obtain ${V}_{s}\approx 0.065\frac{\mu m\,}{s}$ and ${t}_{{\rm{set}}}\approx 38-43\mathrm{h}$. Thus, the estimated bacterial settling time throughout the full height of the biocell is in the range of time required after hardware integration and launch. This calculation does not account for bacteria that were already suspended in median bulk areas, which would have settled even sooner.

The considerations discussed above therefore led to three testable configurations of NemaCapsules: (i) Chambers positioned down on the top rack (sedimentation favored) and the PDMS floor against a narrow gap; (ii) Chambers positioned down on the top rack (sedimentation favored) and the PDMS floor exposed to a larger gap- this was only feasible for 6 of the 12 NemaCapsules; and (iii) Chambers face up on the top rack, increasing distance between settled bacteria and sieve inlets and the PDMS floor exposed to a larger gap.

Since only configurations (i) and (iii) could be used on the top shelf, we compared crawling worm viability across all three conditions (Fig. 3c). WT(N2) was the only strain evaluated in these studies, alongside 2 microbiomes, *E. coli* OP50 and 3-biome. To incorporate technical replicates of each condition in the single PHAB provided we prioritized comparison of NemaCapsules orientations. Worms were scored as viable if movement was observed in full videos of the arena. As expected, configuration (ii) consistently showed >90% viability. Among the alternative configurations, (i) showed clearer viability than (iii), particularly on day 9 in 3-biome fed chambers (2-way ANOVA *p* = 0.0158). These results suggest that aligning bacterial sedimentation toward sieve channels plays a more significant role in supporting viability than maximizing air gap alone. Thus, on the top-row slots, configuration (i) was favored over (iii). We also observed a similar trend for swimming worms (Supplementary Fig. 1). Overall, these studies demonstrated that the PHAB will serve as a robust and viable secondary containment for the NemaCapsules in spaceflight studies **–**especially when utilizing the optimized orientation configurations.

### Viabilitiesof worms with different host/microbiome conditions in NemaCapsules

Changes in host genetic background and microbiome can impact many aspects of host physiology and growth^19^. Thus, we next sought to examine whether NemaCapsules could be robustly applied to cultivate diverse worm strains under a variety of microbiome conditions. To test this, we selected four *C. elegans* host strains [WT(N2), *daf-16*, ED3017, and JU1400] across three microbiome conditions [OP50, 3-biome, and the 63-member BIGbiome] and monitored worm viability over days 5 through day 11.

This range of timepoints was chosen based on observations in previously published work showing that defined microbiome communities establish intestinal colonization over this period, as follows. Studies by Zhang et al. show that BIGbiome gut colonization is not detected until day 2 post exposure and differences in colonization density and composition stabilize by day 5 post exposure^19^. Additionally, CFU studies from Portal-Celhay et al. show that bacteria can colonize at high loads in the adult intestine across mid-adulthood—as much as ~10⁴ CFU/worm by ~day 4 and remains near that level through at least day 8, with persistence reported out to ~day 14^55^. Other previously defined microbiome communities, such as CeMbio, demonstrate that individual adults are consistently colonized around a similar duration of exposure^56^, supporting days 5 to 11 as a suitable range of timepoints to examine viability amongst the varied host strain and microbiome conditions in the NemaCapsules. Colonization was not measured in the present study.

**Fig. 4:**
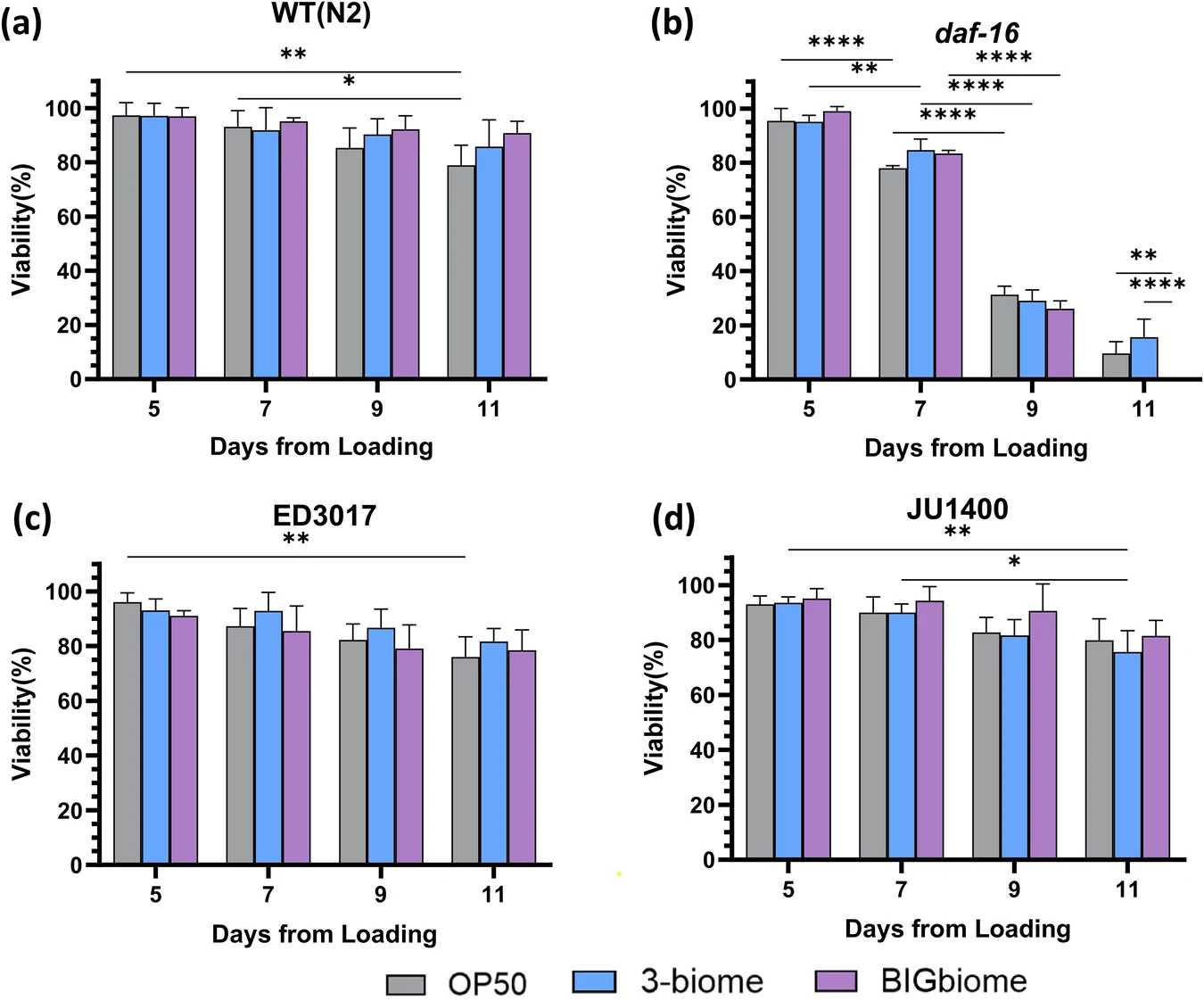
Worm viability examined longitudinally in NemaCapsules across strain and microbiome conditions. 2-way ANOVA used to compare between 3 defined microbiomes and 4 timepoint for each plot. Means and standard deviations were determined from PNC replicates. Significance was denoted by *p* < 0.05, with insignificant comparisons not listed on the plots. **a** WT/OP50 (*N* = 2, *n* = 2 PNCs, 107 animals); WT/3-biome (*N* = 3, *n* = 3 PNCs, 123 animals); WT/BIGbiome (*N* = 3, *n* = 3 PNCs, 140 animals); 2-way ANOVA (*p* = 0.041–0.999). **b** *daf-16*/OP50 (*N* = 2, *n* = 2 PNCs, 99 animals); *daf-16*/3-biome (*N* = 3, *n* = 3 PNCs, 123 animals); *daf-16*/BIGbiome (*N* = 3, *n* = 3 PNCs, 140 animals); 2-way ANOVA (*p* = <0.0001–0.006). **c** ED3017/OP50 (*N* = 2, *n* = 2 PNCs, 91 animals); ED3017/3-biome (*N* = 3, *n* = 3 PNCs, 117 animals); ED3017/BIGbiome (*N* = 3, *n* = 3 PNCs, 145 animals); 2-way ANOVA (*p* = 0.004–0.999). **d** JU1400/OP50 (*N* = 2, *n* = 2 PNCs, 93 animals); JU1400/3-biome (*N* = 3, *n* = 3 PNCs, 123 animals); JU1400/BIGbiome (*N* = 3, *n* = 3 PNCs, 149 animals); 2-way ANOVA (*p* = 0.005–0.999).

In this time window, the viabilities for crawling worms are shown in Fig. 4, while the counterpart values for swimming worms are compiled in Supplementary Fig. 2. The trends among worm strain/microbiome conditions remained consistent between swimming and crawling worms. Also, no statistical significance between microbiomes (within a single timepoint) and most consecutive timepoints (compared within a single microbiome) was observed (2-way ANOVA *p* = 0.0531–0.999). However, significance was observed within OP50 between earliest and latest timepoints— days 5,7 vs. OP50 day 11 (2-way ANOVA *p* = 0.0058–0.041).

In contrast, *daf-16* mutants showed rapid declines in viability, particularly after day 7, dropping well below 40% viability by day 9 for all microbiomes (2-way ANOVA *p* < 0.0001) (Fig. 4b). This decline was expected since *daf-16* mutants are short-lived due to a loss in stress response and longevity genes regulated by insulin signaling pathways^57^. Additionally, *daf-16* worms fed BIGbiome experienced full population death by day 11, before those fed OP50 and 3-biome. Wild isolates ED3017 and JU1400 maintained intermediate viability above 70% through day 9 across most conditions, with significant changes only occurring on day 11, with respect to the same microbiome on days 5 and/or 7 (2-way ANOVA *p* = 0.005-0.041) (Fig. 4c, d). No significance (2-way ANOVA *p* = 0.051-0.999) was observed between microbiomes for viability.

### Assessment of crawling and swimming among host- microbiome conditions

The viability trends observed across the host strains and microbiomes inform on the resilience of different host-microbiome conditions inside the NemaCapsules. However, viability alone does not capture an assessment of functional health^58^. Therefore, we determined locomotion profiles as functional measures for host-microbiome interactions under spaceflight conditions. The NemaCapsules system enables locomotion assessments across two modes (crawling and swimming) under the same microbiome environment, allowing comparative analysis. Here, we focused on days 7 and 9 for imaging to capture both baseline locomotion (day 7) and age-induced shifts (day 9) across host/microbiome conditions.

We quantified crawling through speed measures generated by wrMTrck^59^, while swimming readouts were quantified through thrashing counts. To ensure accuracy, wrMTrck- generated speed measurements were compared with manual measurements from the same videos and showed no significance between distributions (two-tailed *t*-test, *p* = 0.955). Additional validation details, including noise-floor analysis, are provided in Supplementary Fig. 3.

**Fig. 5:**
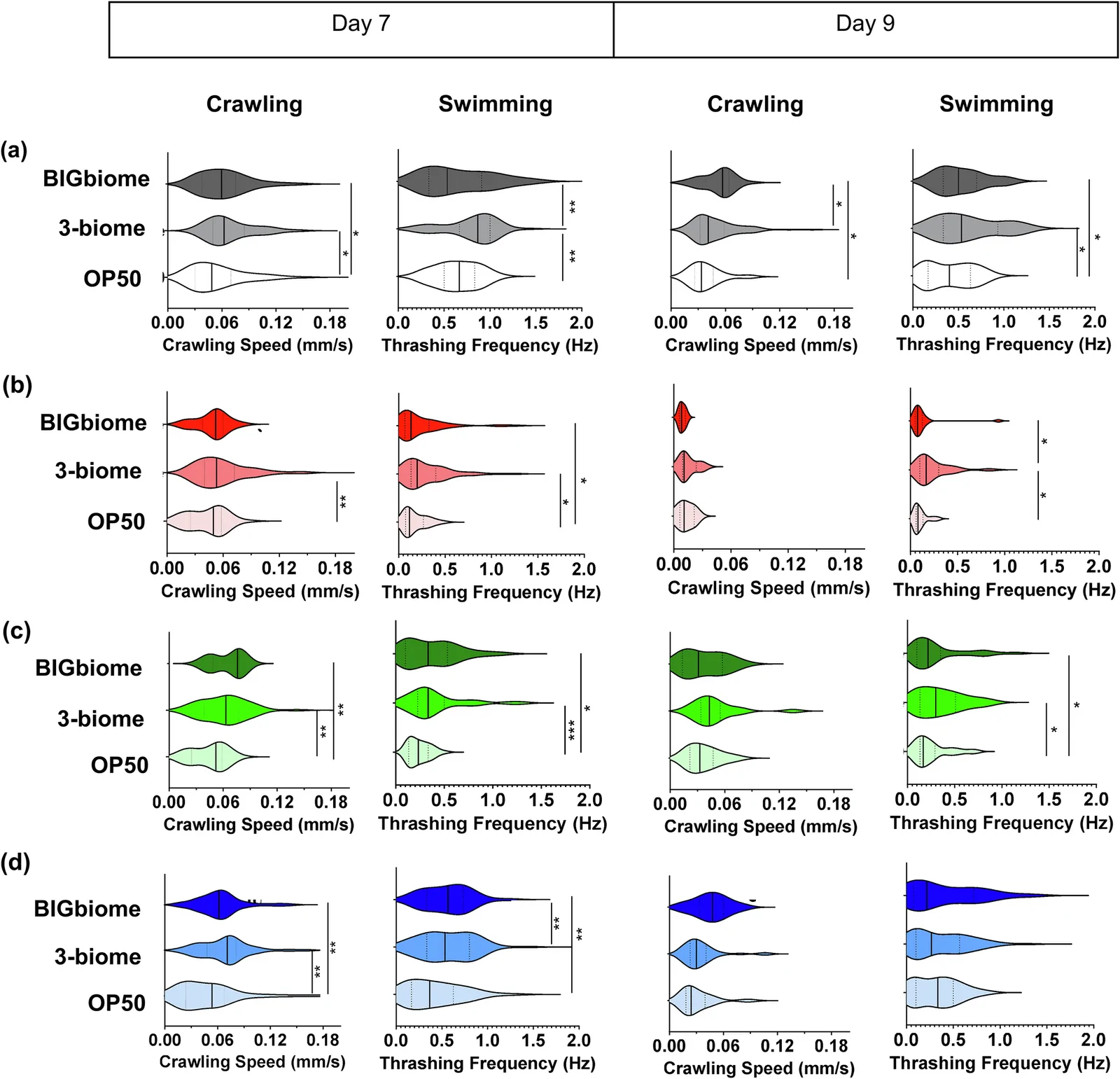
Longitudinal studies of gut microbiome effects within different host strains on days 7 and 9 from loading. Each section consists 2 plots for each host and time point condition: speed for crawling, thrashing for swimming. The violin plots display individual worm values (for visualization purposes only), while statistics are generated from means and standard deviations from PNC chamber replicates. **a** WT(N2) strain microbiome comparison (*N* = 3 trials, *n* = 1 swimming, 1 crawling replicate per trial, 40–50 animals per chamber) Days 7 and 9 from loading. **b** *daf-16* strain microbiome comparison (*N* = 3 trials, *n* = 1 swimming, 1 crawling replicate per trial, 40–50 worms per chamber) Day 7 and 9 from loading; **c** ED3017 strain microbiome comparison (*N* = 3 trials, *n* = 1 swimming, 1 crawling replicate per trial, 40–50 worms per chamber) Days 7 and 9 from loading; **d** JU1400 strain microbiome comparison (*N* = 3 trials, *n* = 1 swimming, 1 crawling replicate per trial, 40–50 worms per chamber) Days 7 and 9 from loading.

**Fig. 6:**
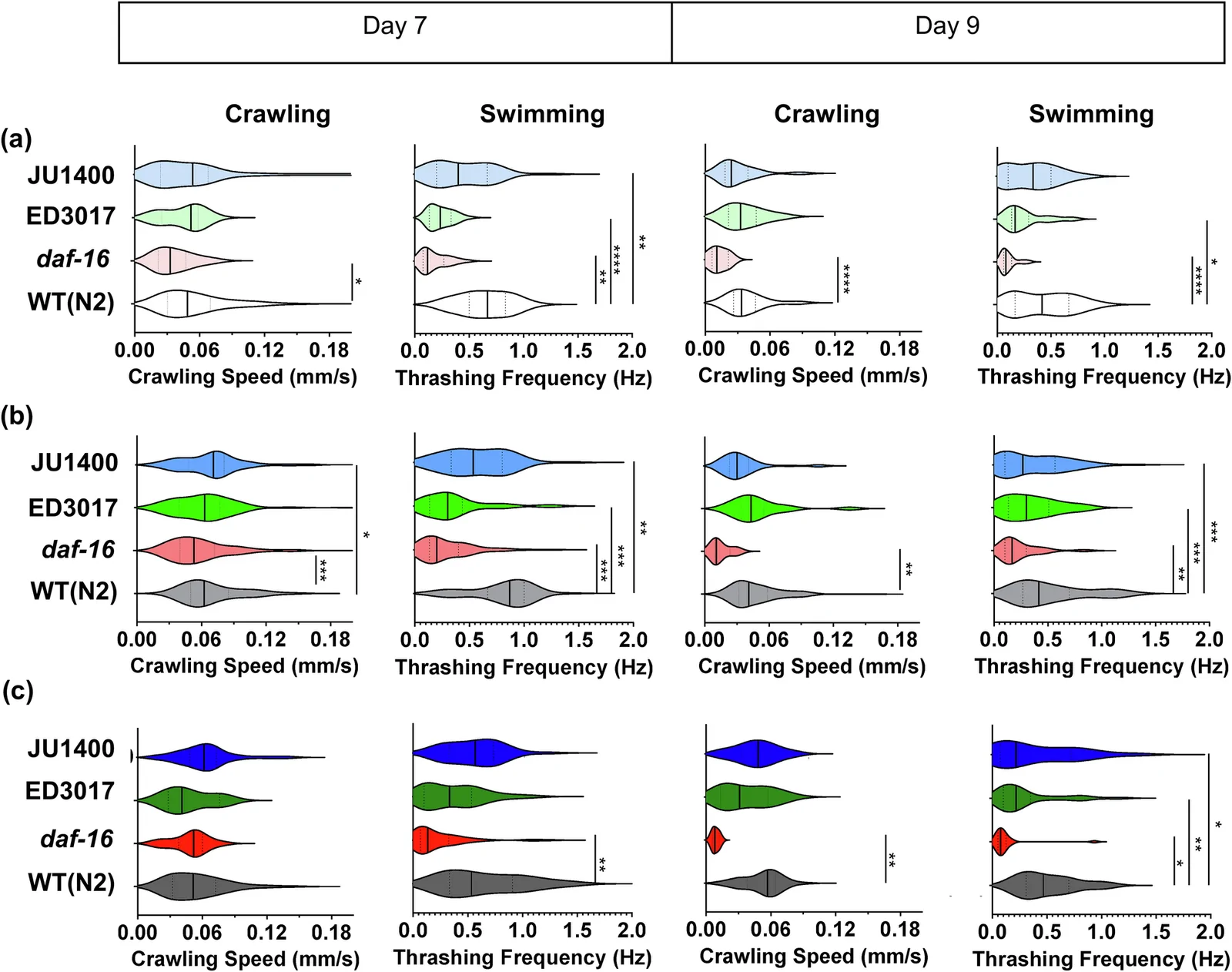
Longitudinal studies of host response effects within different microbiomes on days 7 and 9 from loading. Each section consists 2 plots for each microbiome diet and time point condition: (1) thrashing for swimming (left) (2) speed for crawling (right). The violin plots display individual worm values (for visualization purposes only), while statistics are generated from means and standard deviations from PNC swimming or crawling replicates. **a** OP50 comparison (*N* = 3 trials, *n* = 1 swimming, 1 crawling chamber per trial, 40–50 worms per chamber) Days 7 and 9 from loading. **b** 3-biome microbiome comparison (*N* = 3 trials, *n* = 1 swimming, 1 crawling chamber per trial, 40–50 worms per chamber) Days 7 and 9 from loading. **c** BIGbiome microbiome comparison(*N* = 3 trials, *n* = 1 swimming, 1 crawling chamber per trial) Day 7 and 9 from loading.

We followed this by quantifying locomotion across host-microbiome conditions at day 7 and day 9 post loading with crawl speed and swim-induced thrashing. This study design was longitudinal, where the host/microbiome conditions were followed in the same PNC chambers over those timepoints. Figure 5 visualizes microbiome-dependent differences within each worm host strain, whereas Fig. 6 re-plots to highlight host strain-dependent differences within each microbiome. Together, these plots provide separate microbiome-, host-, and age-dependent effects.

We first examined microbiome-dependent effects within each host strain (Fig. 5). The baseline for statistical comparisons was OP50. WT(N2) showed distinct distributions for both swimming and crawling between OP50 vs. 3- and BIGbiome on day 7 (Fig. 5a). Both multi-membered biomes showed 21 to 44% higher means compared to OP50 (two-tailed *t*-test, *p* = 0.018–0.045). On day 9, WT(N2) exhibited statistically significant differences in 3- and BIGbiome crawling (Fig. 5a) (Kolmogorov–Smirnov test, *p* = 0.002 to 0.045), with continued locomotory enhancement from both multi-membered biomes.

Notably, *daf-16* mutants exhibit heightened sensitivity between microbiomes, with both crawling and swimming scores as follows on day 7—BIGbiome (respective means = 0.049 mm/s,0.23 Hz) ≈ OP50(respective means=0.044 mm/s,0.18 Hz) < 3-biome(respective means=0.060 mm/s,0.29 Hz) in Fig. 5b. Locomotion in the *daf-16* deletion background nearly ceases for both swimming and crawling by day 9, with means declining at least 70% from that of day 7, as expected from the accelerated aging due to the loss in regulation of insulin signaling^60^.

ED3017 and JU1400 demonstrated enhanced means in crawling and swimming under 3-biome and BIGbiome by 23 to 67%, compared to OP50 in both crawling and swimming on day 7 in Fig. 5c, d (two-tailed *t*-test, *p* = 0.002 to 0.005). By day 9, microbiome-driven enhancements compared to OP50 have largely diminished for ED3017 and JU1400. The overall decline reflects consequences of *C. elegans* aging gradually impairing locomotion, which will also hold in flattening out the differences between microbiome treatments. It should be noted that in some day 9 conditions (Fig. 5b, c), microbiome differences that were not detected in crawling were evident in swimming.

Host genotype variation also influenced locomotion patterns within microbiome conditions, as shown in Fig. 6, to allow for direct visualization of host ranking and aging effects across both crawling and swimming. The baseline for comparison here was WT(N2). Within the OP50 microbiome on day 7 (Fig. 6a), *daf-16* exhibited significantly lower thrashing frequency and crawling speed compared to WT(N2) with respectively up to a ≈70 and 30% difference between means, reinforcing known heightened vulnerability in locomotion assays (two-tailed *t*-test, *p* = <0.0001–0.035). *daf-16* mutant deficits were anticipated, given that the deletion of DAF-16/FOXO removed key pathways to insulin signaling, rendering the *daf-16* mutant vulnerable to accelerated aging and impaired locomotion^57,60^. ED3017 and JU1400 exhibited little differences (if at all) in both thrashing and crawling, comparable to WT(N2) (two-tailed *t*-test, *p* = 0.098 to 0.728). By day 9, *daf-16* exhibited severe locomotory decline (two-tailed *t*-test, *p* < 0.0001 to 0.0002) with the mean declining over 70% as compared to day 7. On the other hand, ED3017 (mean=0.037 mm/s) and JU1400 (mean=0.0335 mm/s) showed statistically indistinguishable crawling speed from WT(N2) (mean=0.038 mm/s) (two-tailed *t*-test, *p* = 0.092 to 0.839).

Across 3-biome and BIGbiome microbiome conditions (Fig. 6b, c) on day 7, locomotion was generally reduced relative to the baseline WT(N2), except for JU1400 crawling, with comparable means (two-tailed *t*-tests, *p* = 0.145 to 0.798). ED3017 and WT(N2) also demonstrated comparable crawling locomotory distributions (two-tailed *t*-test, *p* = 0.098 to 0.477). However, in OP50 and 3-biome, significant differences were observed in thrashing data between wild isolates and WT(N2) (two-tailed *t*-test, *p* = <0.0001 to 0.006), which could be hypothesized based on literature to reflect the higher energy of swim movements and distinct mechanisms from crawling speed^40^.

The overall host ranking in locomotory activity was generally *daf-16* as the most limited, followed by ED3017, JU1400, and then WT(N2). WT(N2) showed the most right-leaning distributions among host strains, while not drastically exceeding wild isolates ED3017 and JU1400.

By day 9, locomotion trends converged across strains, with ED3017 and JU1400 crawling showing no significant differences from WT(N2) (*t*-test, *p* = 0.092 to 0.498). However, significant differences were observed between their respective thrashing in 3- and BIGbiome on day 9 (two-tailed *t*-test, *p* = 0.0001 to 0.013). Significance between swimming data of these host strains shows better resolution between locomotory capacity than crawling. These findings suggest that while early locomotion trends reflect genetic and microbiome interactions, long-term locomotory decline appears to be a shared effect across host backgrounds. These findings reinforce the role of *daf-16* as a locomotory stress-sensitive strain, highlight intermediate levels of activity in ED3017 and JU1400, and support WT(N2) as a stable locomotory reference.

Overall, the viability and locomotion datasets shown in this section demonstrate that NemaCapsules support long-term cultures, in addition to yielding biologically expected differences between host-microbiome conditions. High worm viability and measurable crawling and swimming locomotion were observed on days 7 and 9, across the host/microbiome conditions (with the exception of *daf-16* on day 9). NemaCapsules were able to resolve microbiome-dependent enhancements to locomotion, from the 3-biome and BIGbiome communities, relative to OP50. Anticipated locomotory activity rankings were also observed among host genetic strains from lowest to highest: *daf-16* < wild isolates < WT(N2). These biologically coherent patterns provide functional validation that the NemaCapsules hardware has the capability of resolving locomotory patterns between specific host strain-microbiome sets under flight-relevant conditions.

## Discussion

NemaCapsules were developed as modular hardware integrating PNC microfluidic chambers into spaceflight biocells, enabling low-intervention *C. elegans* assays for spaceflight in a flight-relevant format. These ground studies establish the value of the NemaCapsules supporting long-duration cultures and imaging, for later locomotory phenotyping—across four host strains and three microbiome conditions, while enabling paired assessment of crawling speed and swim-induced thrashing within the same sealed culture environment. Collectively, these results demonstrate that the NemaCapsules can resolve functional differences across host genotypes and the defined microbiome conditions.

Across conditions, multi-membered communities (3-biome and BIGbiome) frequently enhanced locomotion compared to OP50. The extent and frequency of these effects varied between host strains, emphasizing that host genetic background is a determinant of microbiome-linked functional outcomes. The results established functional and associative support for the NemaCapsules validation, through baseline microbiome (day 7) and aging effects (day 9), in both crawling and swimming readouts.

These results demonstrate the NemaCapsules’ ability to capture biologically meaningful variation across host-microbiome conditions, at the level of whole-organism phenotypes rather than underlying mechanisms. In this section, we draw upon previous studies for possible explanations that could align with our experimental observations.

The microbiome-dependent locomotion patterns support that defined microbiome conditions can yield distinct outcomes in NemaCapsules. Within the host strains, animals exposed to 3- or BIGbiome demonstrated locomotion distributions distinct from those observed with OP50, especially on day 7. Previous studies provide possible explanations for these patterns. Multi-membered microbial communities maybe provide additional nutrients or other such inputs, not found in OP50 alone. Earlier work on defined *C. elegans* microbiomes has identified members such as *Ochrobactrum*, among others, in enhancement of host health markers, such as development, body size, intestinal colonization, and viability^18,19,61–63^.

Different host genotype conditions also demonstrated different functional responses within microbiome conditions. *daf-16* mutants displayed the strongest decline in viability and locomotory performance, consistent with prior literature linking DAF-16/FOXO to stress resistance and longevity.

The loss of the DAF-16/FOXO gene dysregulates host-microbiome interactions by changing worms' immunity and gut colonization potential^57,60^. This highlights the crucial role of DAF-16/FOXO in maintaining the balance of host-microbiome health, which also manifests in locomotory activity.

In contrast, WT(N2), ED3017, and JU1400 show higher levels of locomotory activity, with mostly WT(N2) being the highest ranked out of the three. One possible literature-informed explanation is that the lab-derived mutations in N2 lie in genes such as *npr-1* that impact foraging and social behavior to gain access to food^64^. The N2 *npr-1* gene mutation, with the 215V substitution, confers more solitary feeding and roaming. This is distinct from the aggregation “social behavior” characteristic of wild isolates containing the 215F NPR-1 variant^65,66^. Our observations are also consistent with prior reports that WT(N2) showed comparable to higher thrashing activity compared to other shorter-lived strains^67^.

A further pattern from the experimental dataset was that some condition-dependent differences were more readily distinguished in swimming than crawling. A possible explanation from prior literature is that swimming and crawling place different demands on the worms^41,42^. This is also consistent with the observation that some day 9 microbiome or host-dependent differences remained more apparent in thrashing than in crawling speed.

Taken together, the platform demonstrated the core characteristics needed for spaceflight studies: (i) passive feeding to reduce crew burden, (ii) an enclosed, full-volume system, (iii) multiplexed chambers for dual locomotory readouts within a single unit, (iv) robust viability across diverse host–microbiome conditions, and (v) feasibility of extracting whole-organismal health phenotypes. Therefore, the ground studies in this work demonstrate that NemaCapsules is well positioned for an upcoming International Space Station study to quantify host–microbiome locomotion responses in spaceflight. In the future flight implementation, NemaCapsules would similarly house the different host and microbiome conditions, contained in the PHAB during incubation periods and removed at the timepoints of interest for video acquisition using the same flight-certified imaging hardware.

The sealed, filled, and minimal-intervention nature of NemaCapsules can also naturally extend to xenobiotic and toxicology testing. It will enable controlled exposure studies both for Earth-based applications and as well as addressing how spaceflight alters organismal resilience to environmental stressors. More broadly, the modularity and minimal-intervention workflow demonstrated here provide a foundation for future multigenerational, behavioral, and microbiome-focused missions. NemaCapsules now offer a scalable route for systematically probing how spaceflight, microbiome, and host genetics interact, with insights relevant to long-duration spaceflight.

## Methods

### *C. elegans* cultures

N2 Bristol (WT(N2)), GR1307 [*daf-16* (mgDf50) I], ED3017 (*C. elegans* wild isolate from Scotland), and JU1400 (*C. elegans* wild isolate from Spain) were the starting cultures. They were rendered progeny-free using an auxin-induced degradation (AID) of an essential sperm protein, SPE-44, via a degron tag mediated by germline-specific expression of P_pie-1_::TIR1 that arrests spermatogenesis^38^. The resulting strains were respectively designated with the following nomenclature: PX627 [fxIs1(Ppie-1::TIR1::mRuby); *spe-44*(fx110[*spe-44*::degron])], BIG0101 [*daf-16*(mgDf50) I; fxIs1(Ppie-1::TIR1::mRuby); *spe-44*(fx110[*spe-44*::degron])], ZB5755 [ED3017; fxIs1(Ppie-1::TIR1::mRuby); *spe-44*(fx110[*spe-44*::degron])], and ZB5756 [JU1400; fxIs1(Ppie-1::TIR1::mRuby); *spe-44*(fx110[*spe-44*::degron])]. The original strains were obtained from the *Caenorhabditis* Genetics Center (CGC).

Mixed-age populations from all strains were cultured on NGM agar plates, each seeded with 500 µL of *E. coli* OP50 bacteria. The mixed population was age-synchronized by extracting eggs from gravid adults using the bleach-based method^8^. Auxin sterilization was adopted as a standard protocol to ensure progeny-free conditions. They were treated 24 h after egg extraction by exposing L1s to plates containing 1 mM IAA (Thermo Scientific 3-Indoleacetic Acid, USA). The auxin plates were made by topically adding 200 µL of 40 mM IAA suspended in M9 to ready-made NGM plates containing 10 mL of the agar mixture.

Worms were loaded into devices at 72 h after hatching (day 1 adults) for both treatments. The day of loading was counted as day 0. The microfluidic PNC chambers were pretreated with 5 wt/v% pluronic solution, followed by aspirating worms suspended in NGM into the devices using a syringe-capillary assembly.

### Bacterial media preparation

Three bacterial conditions were prepared to test all strains: *E. coli* OP50; 3-biome (consisting of a 1:1:1 mixture of the dominant colonizers of the BIGbiome community, *Ochrobactrum* BH3, *Myroides* BIGb0244 and *Lelliotia* JUb66); and BIGbiome. OP50 is a single-membered bacteria that served as the dietary control, whereas 3-biome and BIGbiome are multi-membered bacterial communities representing the wild *C. elegans* microbiome. The Samuel Lab provided suspensions of BIGbiome in S-basal.

OP50 as well as individual members of 3-biome and BIGbiome were inoculated in overnight LB cultures at 37 °C and 26 °C, respectively. OP50 and the 3-biome species were pelleted and resuspended to an OD≈ 1. The 3-biome constituents were combined in equi-volume amounts. Subsequently, the OD-adjusted OP50 and 3-biome mixtures were pelleted and concentrated to 20 mg/mL in NGM. The Samuel lab completed the inoculation, initial OD adjustments and suspension for BIGbiome. The OD≈ 1-adjusted BIGbiome was pelleted and concentrated to 20 mg/mL in NGM.

### Fabrication of PNC chambers

The microfluidic devices were made using casting polydimethylsiloxane (PDMS) in molds fabricated in-lab through UV-based SU-8 soft lithography. A 1.5–2.0 mm thick PDMS (Sylgard 184A and B, 1:10 by weight, Dow Corning, USA) layer was cast in the mold and the inlet holes were punched with a 1 mm biopsy puncher. 2 PDMS devices (1 crawling chamber, 1 swimming chamber) were bonded to a 1 mm PDMS sheet (Bisco® HT6240, Rogers Corporation, NJ) with 1.5 min of plasma bonding (Harrick Plasma, NY), followed by heating them at 70 C with light weights (up to 1 lb.) sitting on top of them for 72 h. Prior to bonding the PDMS sheet substrates are manually shaped to mirror the geometry of the biocell frame holders.

### NemaCapsules assembly and loading

Prior to worm and microbiome loading, the bonded pair of chambers were integrated into the biocells by screwing in the PDMS sheet aligned between the provided structural support and accessories (BioServe Space Technologies ®). The pre-loading treatment consisted of injecting chambers with 5 wt% Pluronic F127 (Sigma-Aldrich) and surrounding them with sterile DI water for at least 20 min to prevent protein and bacterial build-up, as well as releasing trapped air bubbles. Each chamber was loaded with 40–50 worms, followed by injecting the chamber interior with the microbiome media and sealing the worm port with a 5 mm-long blocker pin. After the worms and food were loaded into the device interior, the top surface of the biocell was covered by an FEP membrane and sealed with another frame holder. The rest of the bulk volume was filled with the designated microbiome through a 25-gauge needle and 5- or 10-mL syringe puncturing through the septa of the closed system. All studies were conducted with the same in-chamber and bulk administration of microbiomes, except for swim chamber vs. petri dish comparisons (loaded with M9 for media) in Fig. 2c, to stay consistent with protocols used to obtain benchmarked values. The Petri dish controls were loaded with 500 µL of M9 media with 30–40 worms each.

### Image acquisition and processing

Two-minute videos of each chamber of worms were acquired with a spaceflight-certified camcorder (Canon XF705 4K) at 15X zoom, magnification set at 70 with a resolution of 3840 ×2160 pixels and 23.98 fps. The full FOV was approximately 43 mm × 24 mm—sufficient to fit one full chamber in addition to the part of the frame containing a label, indexing the host and microbiome condition. This resulted in the conversion of 1 pixel ≈13.5 µm. Thus, the worm density per frame was the number of worms per given chamber (see section “NemaCapsules assembly and loading” of “Methods”). The imaging setup consisted of the NemaCapsule positioned in a fixed, side-slitted holder on the benchtop and illuminated from the sides using two LED light sources (Supplementary Fig. 4a). The camera-to-sample and lighting distances were held constant across recordings.

All videos were recorded after equilibrating the PHAB to ambient temperature after leaving it in 21 ± 1 °C for at least 1.5 h, before the start of the imaging period. The recorded videos were processed with the following workflow: (1) conversion from MXF to MP4 using the Wondershare UniConverter 15 software (2) conversion from MP4 to AVI with an offline script written in MATLAB (Mathworks, R2023).

### Analysis

Worms were counted and scored from the converted MP4 videos. The viability analysis was reflective of the percentage of worms scored alive, out of the PNC chamber populations, based on any observed motion. A worm was scored dead if it failed to show any movement during the 2 min duration of the video. The bar plots were generated through GraphPad Prism 10.3.1 (*p*-values reported in figure captions), with statistical significance set at *p* < 0.05.

Locomotion phenotyping from videos has been broadly applied in *C. elegans* behavioral studies, through different tracking platforms in previously published work^68,69^. In this work, swimming and crawling were quantified through different approaches. These data sets were visualized with violin plots generated in GraphPad Prism 10.3.1

Crawling was quantified through speeds obtained with the WrmTrck plugin on Image J^59^, using the pipeline detailed in Supplementary Fig. 4b. For the speed measurements, spatial calibration was required on Image J, using a line selection across a known length (e.g., the worm arena length/width of 15 mm), and then applied to the “Set Scale” function to convert pixel distances to physical units. This calibration was conducted on each individual chamber sample, which carried through to the wrMTrck calculations for speed reported in mm/s. The converted AVI video was background-subtracted and thresholded to binarize the worms before running the plugin with the appropriate parameters, as shown in Supplementary Table 4. The raw output results included the speed-based calculations as well as the thresholded video annotating each worm to a specific output value (Supplementary Fig. 3b). The annotated video and the csv results were for later compilation in violin plots on GraphPad Prism 10.3.1. The labeled binarized videos are saved as AVI videos for later reference if needed.

Swimming was quantified by manually counting the thrashes of individual worms. A single thrash is a complete bending movement of the worm’s head from one side to the other. Thrashes were counted at 3 time points for each video, approximately 40–45 s apart. At each time point, the thrashes per worm were counted for 15 s. This was repeated for 15 worms per time point. Worms in the frame were excluded if they were too clustered for clear, visible counts. It should be noted for the swimming benchmark comparisons that speed and path length of the swimming worms were analyzed in addition to the thrashing—the latter two obtained through wrMTrck, with parameters shown in Supplementary Table 3b.

### Statistical analysis

Statistical analyzes for viability and locomotory data were also conducted on GraphPad Prism 10.3.1, with chambers used as the unit of replication. All tests set significance at *p* < 0.05. Benchmarking comparisons for thrashing, swimming speed, and path length were assessed for statistical trends using two-tailed *t*-tests. For viability analyzes, each plot represented one worm strain, which included 2 variables: microbiome condition and timepoint. Hence, we utilized the 2-way ANOVA tests for viability analyzes within each strain. Locomotion datasets displayed the distributions that culminated from individual worm values. However, their statistical analyzes were also conducted between chamber replicates using two-tailed *t*-tests. Chamber-level summaries are provided in Supplementary Table 2, followed by *p*-values from pairwise comparisons from unpaired 2-tailed *t*-tests in Supplementary Table 3.

## Supplementary information

Supplementary information

Supplementary movie1

Supplementary movie2

## Data Availability

All data generated or analyzed during this study are included in the main text and supplementary information. Other data that support the findings of this study are available from the corresponding author upon reasonable request.

## References

[1] Magne, F., O’Ryan, M. L., Vidal, R. & Farfan, M. The human gut microbiome of Latin America populations: a landscape to be discovered. *Curr. Opin. Infect. Dis.* **29**, 528–37 (2016). 10.1097/QCO.0000000000000300 27479024

[2] Tesei, D., Jewczynko, A., Lynch, A. M. & Urbaniak, C. Understanding the complexities and changes of the astronaut microbiome for successful long-duration space missions. *Life* **12**, 495 (2022). 10.3390/life12040495 PMC9031868 35454986

[3] Voorhies, A. A. et al. Study of the impact of long-duration space missions at the International Space Station on the astronaut microbiome. *Sci. Rep.* **9**, 9911 (2019). 10.1038/s41598-019-46303-8 PMC6616552 31289321

[4] Mehta, S. et al. Multiple latent viruses reactivate in astronauts during Space Shuttle missions. *Brain Behav. Immun.* **41**, 210–7 (2014). 10.1016/j.bbi.2014.05.014 24886968

[5] Hunt, P. R. The C. elegans model in toxicity testing. *J. Appl. Toxicol.* **37**, 50–9 (2017). 10.1002/jat.3357 PMC5132335 27443595

[6] Porta-de-la-Riva, M., Fontrodona, L., Villanueva, A. & Cerón, J. Basic Caenorhabditis elegans methods: synchronization and observation. *JoVE* **64**, e4019 (2012). 10.3791/4019 PMC3607348 22710399

[7] Tejeda-Benitez, L. & Olivero-Verbel, J. *Caenorhabditis elegans*, a biological model for research in toxicology. In *Reviews of Environmental Contamination and Toxicology*, Vol. 237. 1–35 (Springer International Publishing Switzerland, 2016). 10.1007/978-3-319-23573-8_1 26613986

[8] Girard, L. R. et al. WormBook: the online review of Caenorhabditis elegans biology. *Nucleic Acids Res.* **35**, D472–D5 (2007). 10.1093/nar/gkl894 PMC1669767 17099225

[9] Park, H.-E. H., Jung, Y. & Lee, S.-J. V. Survival assays using Caenorhabditis elegans. *Mol. Cells* **40**, 90 (2017). 10.14348/molcells.2017.0017 PMC5339508 28241407

[10] Stiernagle, T. Maintenance of C. elegans. 51–67 (Oxford University Press, 1999).

[11] Ishioka, N. & Higashibata, A. Space experiments using *C. elegans* as a model organism. in *Handbook of Space Pharmaceuticals* 647–77 (Springer, 2022).

[12] Gerbaba, T. K., Green-Harrison, L. & Buret, A. G. Modeling host-microbiome interactions in *Caenorhabditis elegans*. *J. Nematol.* **49**, 348 (2017). PMC5770281 29353922

[13] Kissoyan, K. A. B. et al. Exploring effects of C. elegans protective natural microbiota on host physiology. *Front. Cell. Infect. Microbiol.* **12**, 775728 (2022). 10.3389/fcimb.2022.775728 PMC8884406 35237530

[14] Lynch, C. M., Nagpal, J., Luczynski, P., Neufeld, K.-A. M., Dinan, T. G., Clarke, G. et al. *Germ-Free Animals: A Key Tool in Unraveling How the Microbiota Affects the Brain and Behavior. The Gut-Brain Axis*. 401–54 (Elsevier, 2024).

[15] Cheddadi, R. et al. Intestinal development and gut disease: contributions from the Caenorhabditis elegans Model. *J. Surg. Res*. **311**, 339–350 (2024). 10.1016/j.jss.2024.10.051 39730237

[16] Kumar, A. et al. Caenorhabditis elegans: a model to understand host–microbe interactions. *Cell. Mol. Life Sci.* **77**, 1229–49 (2020). 10.1007/s00018-019-03319-7 PMC11104810 31584128

[17] Andersen, E. C. & Rockman, M. V. Natural genetic variation as a tool for discovery in Caenorhabditis nematodes. *Genetics* **220**, iyab156 (2022). 10.1093/genetics/iyab156 PMC8733454 35134197

[18] Zhang, F. et al. Caenorhabditis elegans as a model for microbiome research. *Front. Microbiol.* **8**, 485 (2017). 10.3389/fmicb.2017.00485 PMC5362939 28386252

[19] Zhang, F. et al. Natural genetic variation drives microbiome selection in the Caenorhabditis elegans gut. *Curr. Biol*. **31**, 2603–18.e9 (2021). 10.1016/j.cub.2021.04.046 PMC8222194 34048707

[20] Nelson, G. et al. Radiation effects in nematodes: results from IML-1 experiments. *Adv. Space Res.* **14**, 87–91 (1994). 10.1016/0273-1177(94)90455-3 11540032

[21] Nelson, G. A. et al. Namatode radiobiology and development in space. Results from IML-1. *Life Sci. Res. Space* **366**, 187–191 (1994).

[22] Szewczyk, N. J. et al. Caenorhabditis elegans survives atmospheric breakup of STS-107, space shuttle Columbia. *Astrobiology* **5**, 690–705 (2005). 10.1089/ast.2005.5.690 16379525

[23] Szewczyk, N. et al. Description of International Caenorhabditis elegans Experiment first flight (ICE-FIRST). *Adv. Space Res.* **42**, 1072–9 (2008). 10.1016/j.asr.2008.03.017 PMC2493420 22146801

[24] Jamal, R. et al. Gene expression changes in space flown Caenorhabditis elegans exposed to a long period of microgravity. *Gravit. Space Res.* **23**, 85–86 (2010).

[25] Honda, Y. et al. Genes down-regulated in spaceflight are involved in the control of longevity in Caenorhabditis elegans. *Sci. Rep.* **2**, 487 (2012). 10.1038/srep00487 PMC3390002 22768380

[26] Sudevan, S. et al. Nematode muscles project in spaceflight experiment. *Biol. Sci. Space* **32**, 6–10 (2018).

[27] Sudevan, S. et al. Loss of physical contact in space alters the dopamine system in C. elegans. *Iscience* **25**, 103762 (2022). 10.1016/j.isci.2022.103762 PMC8810405 35141505

[28] Soni, P. et al. Microfluidics-integrated spaceflight hardware for measuring muscle strength of *Caenorhabditis elegans* on the International Space Station. *npj Microgravity* **8**, 50 (2022). 10.1038/s41526-022-00241-4 PMC9640571 36344513

[29] Soni, P. et al. Spaceflight induces strength decline in *Caenorhabditis elegans*. *Cells* **12**, 2470 (2023). 10.3390/cells12202470 PMC10605753 37887314

[30] Singh, A. & Luallen, R. J. Understanding the factors regulating host–microbiome interactions using *Caenorhabditis elegans*. *Philos. Trans. R. Soc. B* **379**, 20230059 (2024). 10.1098/rstb.2023.0059 PMC10945399 38497260

[31] Shapira, M. Host–microbiota interactions in Caenorhabditis elegans and their significance. *Curr. Opin. Microbiol.* **38**, 142–7 (2017). 10.1016/j.mib.2017.05.012 28623729

[32] Zhang, J., Holdorf, A. D. & Walhout, A. J. C. elegans and its bacterial diet as a model for systems-level understanding of host–microbiota interactions. *Curr. Opin. Biotechnol.* **46**, 74–80 (2017). 10.1016/j.copbio.2017.01.008 PMC5544573 28189107

[33] San-Miguel, A. & Lu, H. Microfluidics as a tool for *C. elegans* research. in *WormBook: The Online Review of C. elegans Biology* (2018). 10.1895/wormbook.1.162.1 PMC4781173 24065448

[34] Kamili, F. & Lu, H. Recent advances and trends in microfluidic platforms for *C.* elegans biological assays. *Annu. Rev. Anal. Chem.* **11**, 245–264 (2018). 10.1146/annurev-anchem-061417-125604 29894230

[35] Rahman, B., Soni, P., Saroyia, A.P., Schenkenfelder, W. & Vanapalli, S.A. Microfluidic Well Plates Integrated with Passive Nematode Culture Chambers for Multiplexed Chemical Toxicity Assays in C. elegans. *Lab on a Chip* **26**, 3083–3101 (2026). 10.1039/d5lc01165e 41979381

[36] Zhao, Y. et al. Changes to social feeding behaviors are not sufficient for fitness gains of the *Caenorhabditis elegans* N2 reference strain. *eLife* **7**, e38675 (2018). 10.7554/eLife.38675 PMC6224195 30328811

[37] Mok, C. et al. High-throughput phenotyping of infection by diverse microsporidia species reveals a wild *C. elegans* strain with opposing resistance and susceptibility traits. *PLoS Pathog.* **19**, e1011225 (2023). 10.1371/journal.ppat.1011225 PMC10030041 36893187

[38] Kasimatis, K. R., Moerdyk-Schauwecker, M. J. & Phillips, P. C. Auxin-mediated sterility induction system for longevity and mating studies in *Caenorhabditis elegans*. *G3 Genes Genomes Genetics* **8**, 2655–62 (2018). 10.1534/g3.118.200278 PMC6071612 29880556

[39] Zhang, L., Ward, J. D., Cheng, Z. & Dernburg, A. F. The auxin-inducible degradation (AID) system enables versatile conditional protein depletion in *C. elegans*. *Development* **142**, 4374–84 (2015). 10.1242/dev.129635 PMC4689222 26552885

[40] Vidal-Gadea, A. et al. *Caenorhabditis elegans* selects distinct crawling and swimming gaits via dopamine and serotonin. *Proc. Natl. Acad. Sci. USA* **108**, 17504–9 (2011). 10.1073/pnas.1108673108 PMC3198358 21969584

[41] Butler, V. J. et al. A consistent muscle activation strategy underlies crawling and swimming in *Caenorhabditis elegans*. *J. R. Soc. Interface* **12**, 20140963 (2015). 10.1098/rsif.2014.0963 PMC4277086 25551155

[42] Laranjeiro, R., Harinath, G., Burke, D., Braeckman, B. P. & Driscoll, M. Single swim sessions in C. elegans induce key features of mammalian exercise. *BMC Biol.* **15**, 1–17 (2017). 10.1186/s12915-017-0368-4 PMC5385602 28395669

[43] Markov, D. A., Lillie, E. M., Garbett, S. P. & McCawley, L. J. Variation in diffusion of gases through PDMS due to plasma surface treatment and storage conditions. *Biomed. Microdevices* **16**, 91–6 (2014). 10.1007/s10544-013-9808-2 PMC3945670 24065585

[44] Yao, M. et al. Mixing and delivery of multiple controlled oxygen environments to a single multiwell culture plate. *Am. J. Physiol. Cell Physiol.* **315**, C766–C75 (2018). 10.1152/ajpcell.00276.2018 PMC6293053 30183322

[45] Blöbaum, L., Täuber, S. & Grünberger, A. Protocol to perform dynamic microfluidic single-cell cultivation of *C. glutamicum*. *STAR Protoc.* **4**, 102436 (2023). 10.1016/j.xpro.2023.102436 PMC10425941 37543944

[46] Liu, Y. et al. Collapse of arbitrary-shaped soft microfluidics. *Int. J. Solids Struct.* **252**, 111821 (2022).

[47] Majmudar, T., Keaveny, E. E., Zhang, J. & Shelley, M. J. Experiments and theory of undulatory locomotion in a simple structured medium. *J. R. Soc. Interface* **9**, 1809–23 (2012). 10.1098/rsif.2011.0856 PMC3385758 22319110

[48] Park, S. et al. Enhanced Caenorhabditis elegans locomotion in a structured microfluidic environment. *PLoS ONE* **3**, e2550 (2008). 10.1371/journal.pone.0002550 PMC2430527 18575618

[49] Hewitt, J. E. et al. Muscle strength deficiency and mitochondrial dysfunction in a muscular dystrophy model of Caenorhabditis elegans and its functional response to drugs. *Dis. Models Mech.* **11**, dmm036137 (2018). 10.1242/dmm.036137 PMC6307913 30396907

[50] Anupom, T. & Vanapalli, S. A. A compact imaging platform for conducting C. elegans phenotypic assays on Earth and in spaceflight. *Life* **13**, 200 (2023). 10.3390/life13010200 PMC9862956 36676149

[51] Sun, M., Khan, Z. S. & Vanapalli, S. A. Blood plasma separation in a long two-phase plug flowing through disposable tubing. *Lab Chip* **12**, 5225–30 (2012). 10.1039/c2lc40544j 23114925

[52] Shiomi, D., Mori, H. & Niki, H. Genetic mechanism regulating bacterial cell shape and metabolism. *Commun. Integr. Biol.* **2**, 219–20 (2009). 10.4161/cib.2.3.7930 PMC2717524 19641734

[53] Baldwin, W. W., Myer, R., Kung, T., Anderson, E. & Koch, A. L. Growth and buoyant density of *Escherichia coli* at very low osmolarities. *J. Bacteriol.* **177**, 235–7 (1995). 10.1128/jb.177.1.235-237.1995 PMC176578 7798137

[54] Moreira, R., Chenlo, F. & Pereira, G. Viscosities of ternary aqueous solutions with glucose and sodium chloride employed in osmotic dehydration operation. *J. Food Eng.* **57**, 173–7 (2003).

[55] Portal-Celhay, C., Bradley, E. R. & Blaser, M. J. Control of intestinal bacterial proliferation in regulation of lifespan in Caenorhabditis elegans. *BMC Microbiol.* **12**, 49 (2012). 10.1186/1471-2180-12-49 PMC3342110 22452899

[56] Dirksen, P. et al. CeMbio-The Caenorhabditis elegans microbiome resource. *G3 Genes Genomes Genetics* **10**, 3025–39 (2020). 10.1534/g3.120.401309 PMC7466993 32669368

[57] Tissenbaum, H. A. DAF-16: FOXO in the Context of C. elegans. *Curr. Top. Dev. Biol.* **127**, 1–21 (2018). 10.1016/bs.ctdb.2017.11.007 29433733

[58] Bansal, A., Zhu, L. J., Yen, K. & Tissenbaum, H. A. Uncoupling lifespan and healthspan in Caenorhabditis elegans longevity mutants. *Proc. Natl. Acad. Sci. USA* **112**, E277–E86 (2015). 10.1073/pnas.1412192112 PMC4311797 25561524

[59] Nussbaum-Krammer, C. I., Neto, M. F., Brielmann, R. M., Pedersen, J. S. & Morimoto, R. I. Investigating the spreading and toxicity of prion-like proteins using the metazoan model organism C. elegans. *J. Vis. Exp.* **95**, 52321 (2015). 10.3791/52321 PMC4354510 25591151

[60] Wang, H., Webster, P., Chen, L. & Fisher, A. L. Cell-autonomous and non-autonomous roles of daf-16 in muscle function and mitochondrial capacity in aging C. elegans. *Aging* **11**, 2295 (2019). 10.18632/aging.101914 PMC6520005 31017874

[61] Ortiz, A., Vega, N. M., Ratzke, C. & Gore, J. Interspecies bacterial competition regulates community assembly in the C. elegans intestine. *ISME J.* **15**, 2131–45 (2021). 10.1038/s41396-021-00910-4 PMC8245486 33589765

[62] Haçariz, O., Viau, C., Karimian, F. & Xia, J. The symbiotic relationship between Caenorhabditis elegans and members of its microbiome contributes to worm fitness and lifespan extension. *BMC Genomics* **22**, 364 (2021). 10.1186/s12864-021-07695-y PMC8136213 34011272

[63] Cassidy, L. et al. The Caenorhabditis elegans proteome response to naturally associated microbiome members of the genus Ochrobactrum. *Proteomics* **18**, 1700426 (2018). 10.1002/pmic.201700426 29513928

[64] Lee, H. et al. Genetic variants that modify neuroendocrine gene expression and foraging behavior of *C. elegans*. *Sci. Adv.* **10**, eadk9481 (2024). 10.1126/sciadv.adk9481 PMC11168454 38865452

[65] Reddy, K. C., Hunter, R. C., Bhatla, N., Newman, D. K. & Kim, D. H. Caenorhabditis elegans NPR-1–mediated behaviors are suppressed in the presence of mucoid bacteria. *Proc. Natl. Acad. Sci. USA* **108**, 12887–92 (2011). 10.1073/pnas.1108265108 PMC3150904 21768378

[66] Weber, K. P. et al. Whole genome sequencing highlights genetic changes associated with laboratory domestication of *C. elegans*. *PLoS ONE* **5**, e13922 (2010). 10.1371/journal.pone.0013922 PMC2978686 21085631

[67] Zhang, H., Gao, S. & Chen, W. Automated recognition and analysis of head thrashes behavior in *C. elegans*. *BMC Bioinform.* **23**, 87 (2022). 10.1186/s12859-022-04622-0 PMC8903547 35255825

[68] Swierczek, N. A., Giles, A. C., Rankin, C. H. & Kerr, R. A. High-throughput behavioral analysis in C. elegans. *Nat. Methods* **8**, 592–8 (2011). 10.1038/nmeth.1625 PMC3128206 21642964

[69] Yemini, E., Jucikas, T., Grundy, L. J., Brown, A. E. & Schafer, W. R. A database of C. elegans behavioral phenotypes. *Nat. Methods* **10**, 877 (2013). 10.1038/nmeth.2560 PMC3962822 23852451

