# Supplementary material for: NemaCapsules: microfluidics-integrated biocells for investigating C. elegans host-microbiome interactions in spaceflight
Source: NPJ Microgravity. 2026 Jul 23;12:101. doi: 10.1038/s41526-026-00634-9 (PMC13759501; doi:10.1038/s41526-026-00634-9)
Supplement: Supplementary file 1 [file 41526_2026_634_MOESM1_ESM.pdf]

(a)

	Mean	Std Dev	p-value	η^2	95% CI
Swim Chamber	1.53	0.33	0.09	0.0314	-0.21 to 0.015
Petri Dish	1.44	0.166			

(b)

	Mean	Std Dev	p-value	η^2	95% CI
Swim Chamber	0.33	0.05	0.09	0.004	-0.05 to 0.003
Petri Dish	0.29	0.03			

(c)

	Mean	Std Dev	p-value	η^2	95% CI
Swim Chamber	5.38	0.8	0.56	0.0005	-0.33 to 0.60
Petri Dish	4.94	0.28			

SI Table 1. Summary statistics for benchmark locomotion measurements in swim chamber and petri-dish conditions, all evaluated on a technical replicate level (i.e no. chambers and no. petri dishes), with n=3 . (a) thrashing frequency, (b) wrMTrck-derived centroid swimming speed, and (c) path length, with condition-specific means and standard deviations together with the corresponding statistical comparison, confidence interval, and effect-size estimate.

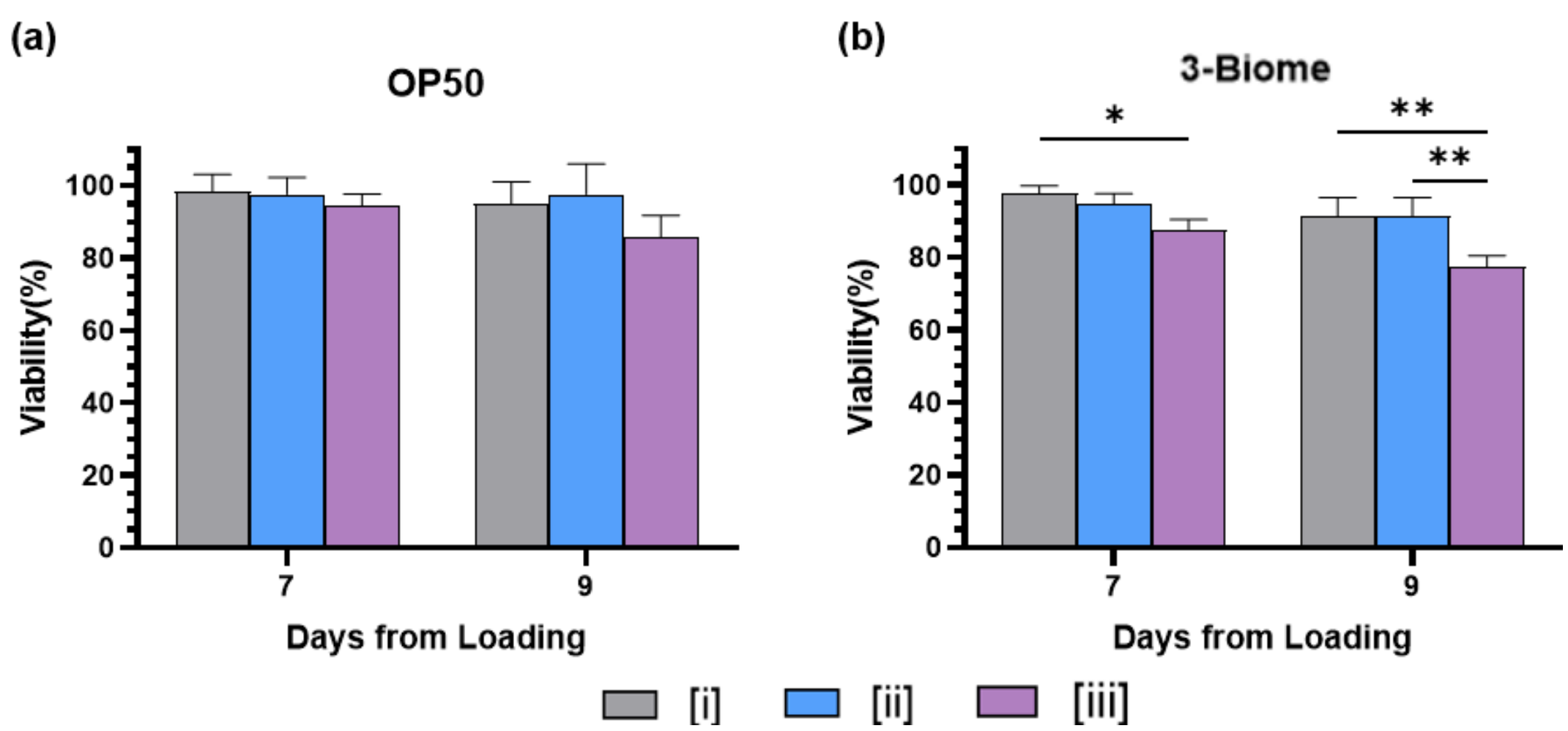

SI Fig 1. PHAB orientation studies for swim chamber worms for WT(N2) worms with N=1 trial, n=2 technical replicates per trial, 40-50 worms, for Days 7 & 9. (a) OP50-fed worms **(b)** 3-Biome-fed worms

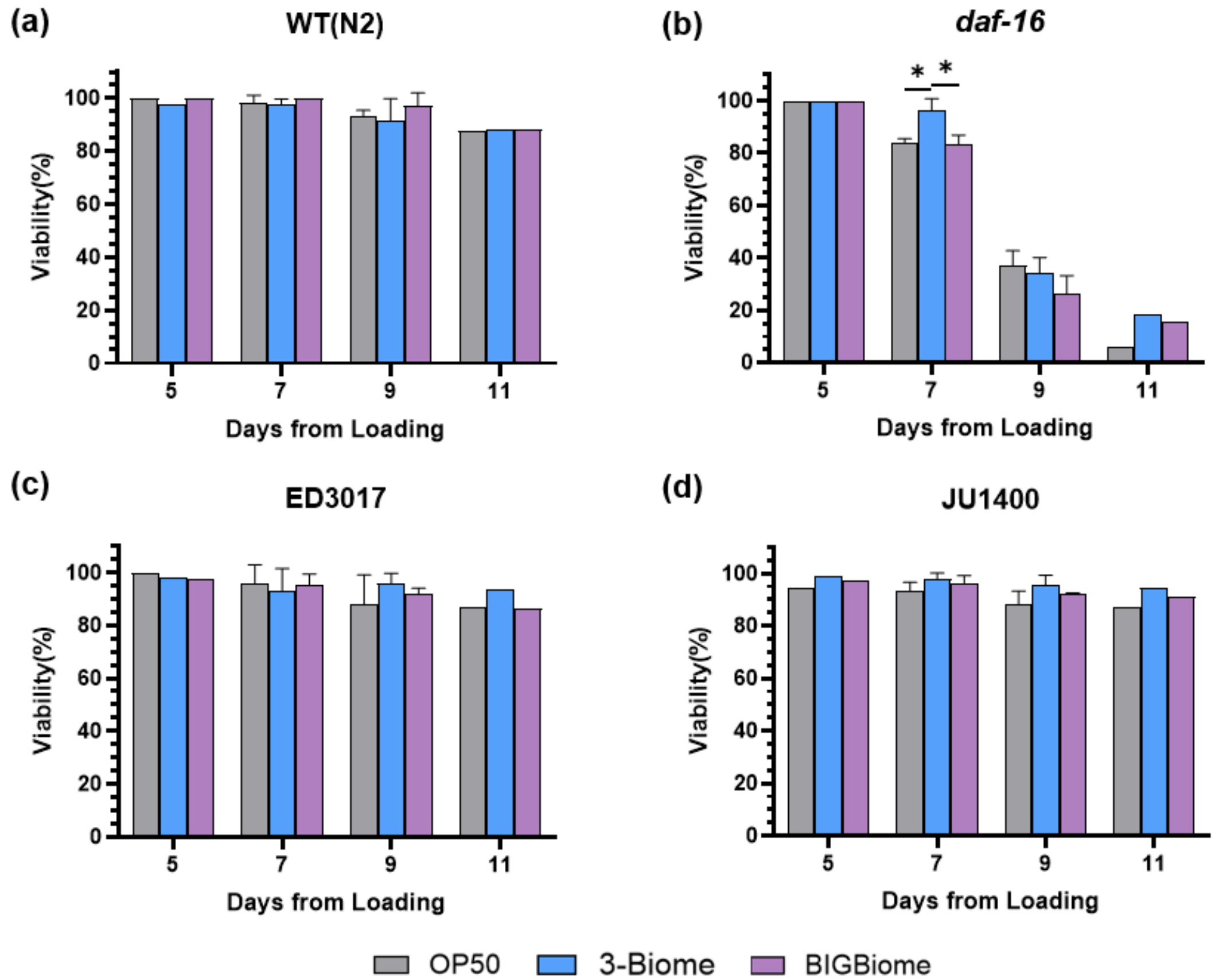

Fig S2. Worm viability in swimming chambers across host strains. Each PNC chamber was loaded with approximately 40-50 worms, with n=1 technical replicate per trial, N=3 trials for Days 7 &9 and N=1 for Days 5 &11(a) WT(N2) **(b)** *daf-16* mutant**(c)** ED3017 **(d)** JU1400

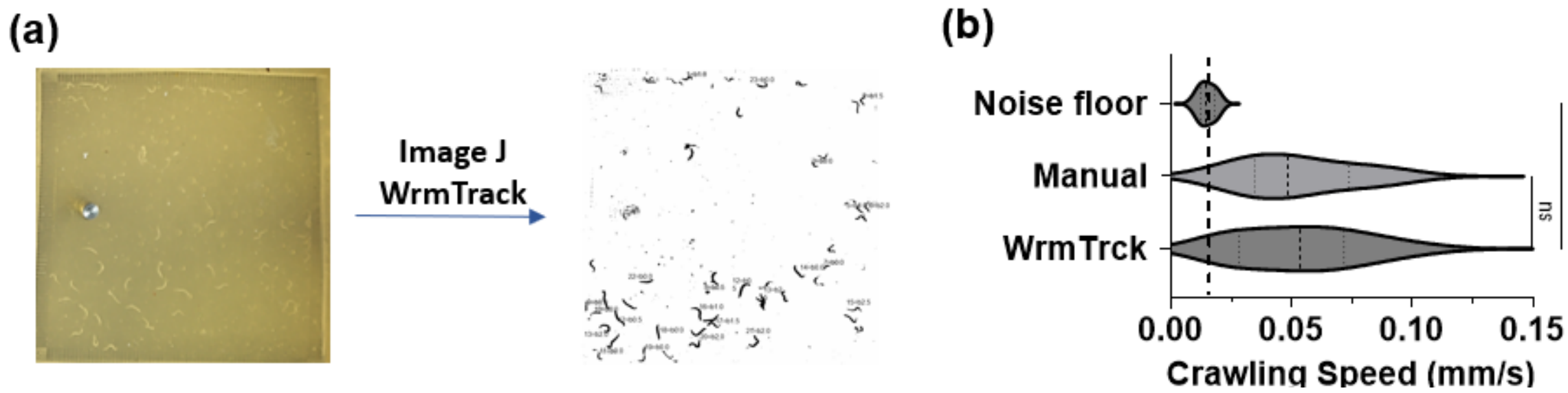

Fig. S3. Imaging to speed calculation frames and noise floor (a) Image of crawling worms from flight-like imaging source Canon XF705 (left-white against brown background) and binarized worms(right-black against white background) thresholded through Image J and run through wrMTrck plugin for speed values (right) **(b)** Comparisons between the wrMTrck, manually calculated animal speeds, as well as artificial speed values generated by the program when run on a dead-worm population (ethanol-killed). For all three, the conditions were WT(N2),3-biome, day 10 from loading. wRMTrck-generated calculation vs. manual showed no significance at $p=0.955$(two-tailed t-test). The noise floor was established at its median speed of 0.015 mm/s—showing minimal overlap with the manual and wrMTrck-generated speeds ($p<0.0001$, two-tailed t-test Test)

		OP50		3-Biome		BIGBiome	
	Days from Loading	Crawl Speed (mm/s)	Thrash Frequency (Hz)	Crawl Speed (mm/s)	Thrash Frequency (Hz)	Crawl Speed (mm/s)	Thrash Frequency (Hz)
WT(N2)	7	0.052±0.002	0.64±0.02	0.075±0.003	0.80±0.05	0.063±0.001	0.65±0.12
	9	0.038±0.001	0.40±0.06	0.048±0.003	0.58±0.065	0.053±0.010	0.52±0.03
daf-16	7	0.044±0.004	0.18±0.04	0.060±0.009	0.29±0.04	0.049±0.011	0.23±0.02
	9	0.012±0.001	0.10±0.03	0.016±0.011	0.26±0.04	0.008±0.001	0.17±0.14
ED3017	7	0.048±0.006	0.33±0.05	0.063±0.004	0.41±0.003	0.061±0.003	0.42±0.18
	9	0.037±0.004	0.29±0.10	0.052±0.026	0.34±0.08	0.044±0.002	0.35±0.02
JU1400	7	0.045±0.006	0.44±0.03	0.08±0.002	0.61±0.03	0.068±0.002	0.53±0.05
	9	0.033±0.004	0.30±0.03	0.033±0.011	0.36±0.05	0.044±0.002	0.39±0.04

SI Table 2. Means and standard deviations of crawling speed and thrashing frequency between 3 technical (chamber) replicates per condition for all the host microbiome conditions in Fig. 5 and 6.

(a)

WT(N2)		0.044	0.045		0.018	0.704
daf-16		0.003	0.862		0.014	0.038
ED3017		0.005	0.003		0.0008	0.018
JU1400		0.002	0.002		0.002	0.001
	OP50	3-Biome	BIGBiome	OP50	3-Biome	BIGBiome
	Crawling			Swimming		

$p > 0.05$
$p < 0.05$ ↑ control
$p < 0.05$ ↓ control

(b)

WT(N2)		0.008	0.704		0.024	0.046
daf-16		0.497	0.242		0.010	0.721
ED3017		0.552	0.749		0.018	0.015
JU1400		0.22	0.20		0.447	0.292
	OP50	3-Biome	BIGBiome	OP50	3-Biome	BIGBiome
	Crawling			Swimming		

(c)

WT(N2)						
daf-16	0.035	0.0009	0.098	<0.0001	0.0002	0.004
ED3017	0.272	0.098	0.477	0.0006	0.0002	0.144
JU1400	0.121	0.798	0.145	0.0006	0.006	0.193
	OP50	3-Biome	BIGBiome	OP50	3-Biome	BIGBiome
	Crawling			Swimming		

(d)

WT(N2)						
daf-16	<0.0001	0.007	0.001	0.001	0.002	0.014
ED3017	0.498	0.839	0.168	0.189	0.0002	0.001
JU1400	0.133	0.092	0.437	0.054	0.0001	0.013
	OP50	3-Biome	BIGBiome	OP50	3-Biome	BIGBiome
	Crawling			Swimming		

SI Table 3. P-values from unpaired two-tailed t-tests with respect to control for both microbiome (OP50 control) and host (WT(N2) control) effects. Green- and pink-highlighted cells stand for respectively higher and lower leaning distributions compared to control. Based on means, standard deviation and 3 technical (chamber) replicates per condition, with significance set at p<0.05 (a) Microbiome comparisons day 7 and **(b)** day 9 **(c)** Host comparisons day 7 and **(d)** day 9

(a)

Segmentation Method	Threshold Method	Maximum Velocity (pixels per frame)	Maximum Area Change (%)	Minimum Object Size (pixels^2)	Maximum Object Size(pixels^2)	Minimum Track Length (frames)
Image Calculator→ Subtraction	Triangle	10	30	90	250	50

(b)

Segmentation Method	Threshold Method	Maximum Velocity (pixels per frame)	Maximum Area Change (%)	Minimum Object Size (pixels^2)	Maximum Object Size(pixels^2)	Minimum Track Length (frames)
Image Calculator→ Subtraction	Triangle	45-60	70-100	90	2000	50

SI Table 4. Methods and parameter value inputs to ImageJ for wrMTrack-based calculations of speed of swimming from videos, for benchmarking in clear media. This includes preprocessing steps: segmentation and thresholding, followed by parameters: object (worm) size limits, maximum velocity (frame-to-frame linking displacement), maximum permitted area change, and minimum retained track length.

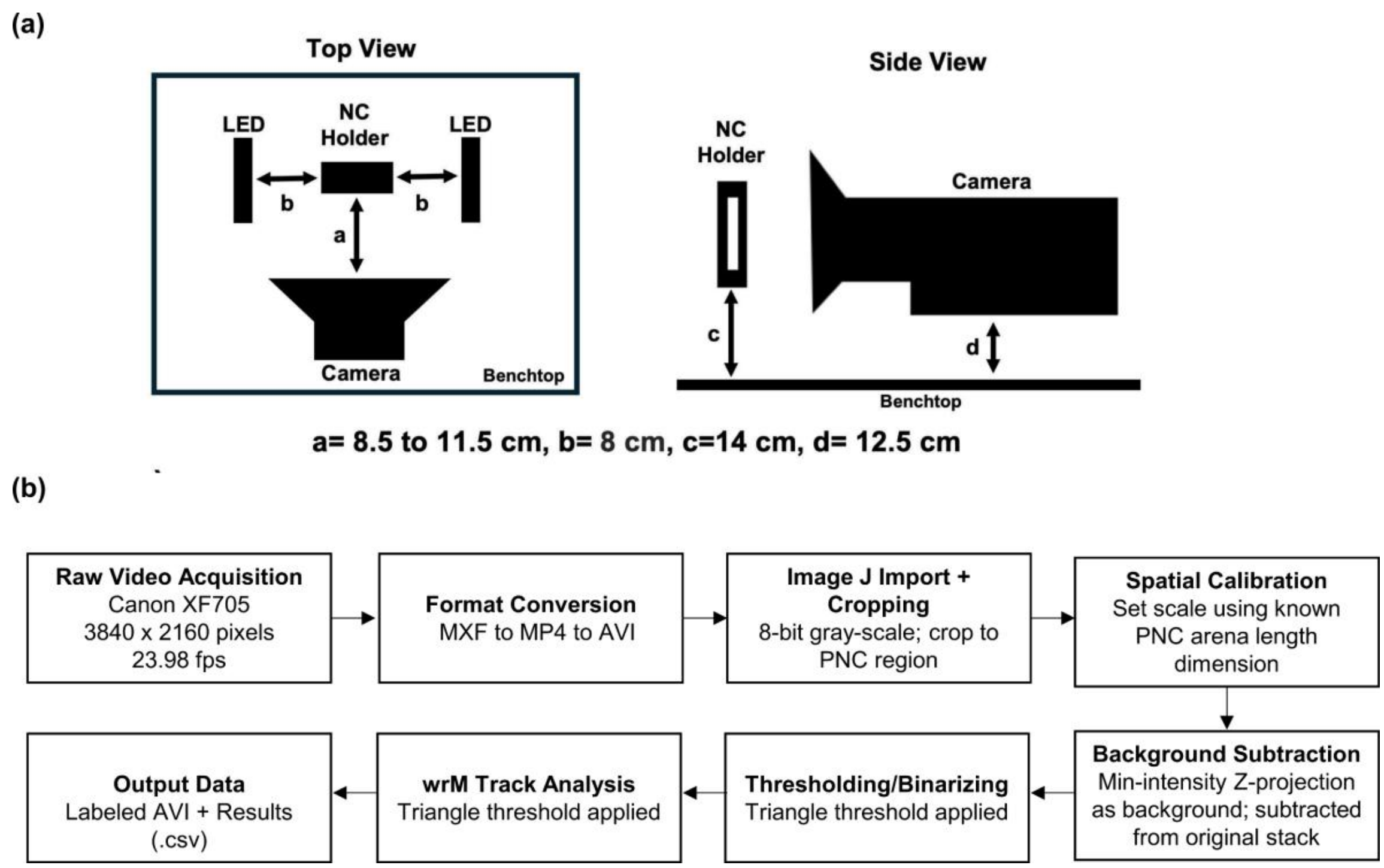

SI Fig. 4. Imaging setup (a) Top and side view of setup positioning NemaCapsule (NC) unit in its holder, in front of the camera lens at 8.5-11.5 cm, and in between 2 led lights equidistantly 8 cm apart **(b)** Pipeline detailing the protocol from raw video conversion to ImageJ processing and wrM Trck calculations and inputs

SI Movie 1. *C. elegans* WT (N2) crawling behavior on OP50 microbiome, Day 7 post-loading. Recorded in NemaCapsules crawling chamber. Worms exhibit coordinated sinusoidal locomotion typical of healthy adults under baseline microbiome conditions, with the speed as the readout.

SI Movie 2. *C. elegans* WT (N2) swimming behavior on OP50 microbiome, Day 7 post-loading. Captured in NemaCapsules swimming chamber. Thrashing frequency is the readout reflecting locomotory activity.
